# Logical computation with self-assembling electric circuits

**DOI:** 10.1371/journal.pone.0278033

**Published:** 2022-12-07

**Authors:** Rojoba Yasmin, Russell Deaton

**Affiliations:** 1 Department of Electrical Engineering, University of Wisconsin Green Bay, Green Bay, WI, United States of America; 2 Department of Electrical and Computer Engineering, The University of Memphis, Memphis, TN, United States of America; Institut Jean Lamour, FRANCE

## Abstract

Inspired by self-assembled biological growth, the Circuit Tile Assembly Model (cTAM) was developed to provide insights into signal propagation, information processing, and computation in bioelectric networks. The cTAM is an abstract model that produces a family of circuits of different sizes that is amenable to exact analysis. Here, the cTAM is extended to the Boolean Circuit Tile Assembly Model (bcTAM) that implements a computationally complete set of Boolean gates through self-assembled and self-controlled growth. The proposed model approximates axonal growth in neural networks and thus, investigates the computational capability of dynamic biological networks, for example, in growing networks of axons. Thus, the bcTAM models the effect of electrical activity on growth and shows how that growth might implement Boolean computations. In this sense, given a set of input voltages, the bcTAM is a system that is able to monitor and make decisions about its own growth.

## Introduction

Distributions of electric potentials in bioelectric networks influence gene expression, and thus, the development of complex biological patterns [1–4]. This feedback between bioelectric and biomolecular mechanisms is postulated to be an ancient mechanism and operates in many cellular processes, including embryonic growth and morphological differentiation [5–7]. Electric signals are also the basis for both communication and computation in neural networks. In this paper, a simple circuit model for growth processes that are influenced by electric potentials, the *Circuit Tile Assembly Model (cTAM)* [8, 9], is extended to implement a computationally complete set of Boolean logic gates in the *Boolean Circuit Tile Assembly Model (bcTAM)*. Thus, the bcTAM informs not only the electrically-influenced growth process, but also how growth can result in computation.

Self-assembly is a model inspired by biological growth in which a larger, more capable system is constructed from smaller components through localized interaction. In the cTAM, larger circuits are self-assembled from unit tiles consisting of basic electrical components. An electric potential drives growth by activating glues to which new tiles bind. As growth proceeds, the potential dissipates, eventually falling below a predefined threshold value, where growth ceases. Thus, the electric potential acts similarly to a finite nutrient supply in a bacterial colony, or for that matter, the electric potential in artificial growth processes, like electroplating [10]. Though a nonbiological system, the cTAM achieves life-like properties, such as self-assembled, self-controlled growth [8, 9, 11–13], and self-replication [14]. Also, the cTAM model is a dynamic model where glues activate when certain criteria are fulfilled. This property is similar to the signal-passing tile assembly model introduced by Padilla, *et al*. [15].

In 1952, Hodgkin and Huxley described how action potentials are initiated and propagated with an equivalent circuit model [16]. The ladder circuits in the cTAM closely resemble those for the propagation of action potentials down axons. This relationship has been more fully explored in [11]. Thus, the bioelectric network that the bcTAM most closely resembles are networks of axons whose growth is influenced by active electric signals, which can elongate axons and change the growth dynamics [17], are potentially important in neural development [17–19], and when coupled with gene expression, have a fundamental role in the growth and organization of neural networks [18]. Our previous works [8, 9, 11–14] investigated electric signal propagation and its impact on dynamic circuit configurations, and this work focuses on the capacity for logical decision-making in the Circuit Tile Assembly Model. The bcTAM model shows the capability of performing Boolean functions in simple, biological mechanisms, such as axon growth. Using different computational models, others have shown the computational power of axons [20], but without growth.

In this paper, the bcTAM model and its working principles and growth mechanisms are defined. Growth is essential to the functionality of the Boolean gates defined in the bcTAM, with growth providing different connections that activate one state of a gate or another. In the abstract, this is similar to conformal changes that produce different functionality in molecular biology. The bcTAM system is able to use logic to reason about its own growth or lack thereof. This extends to Boolean satisfiability problems and a version of bcTAM growth that is NP-complete, demonstrating the complexity of which it is capable.

## Introducing Boolean Circuit Tile Assembly Model (bcTAM)

Biological organisms control molecular self-assembly using biochemical circuits and algorithms [21]. Motivated by these mechanisms, the *Circuit Tile Assembly Model* combines chemically-inspired glues and electric circuitry. The basic cTAM is a self-controlled self-assembly model [8, 9], and achieves self-replication with modified electric circuit components in the *Replicating Circuit Tile Assembly Model (rcTAM)* [14]. Capacitors and time-varying signals are incorporated into the cTAM in [11], termed the *Axonal cTAM (acTAM)*, in which the exact response and signal propagation of the network is calculated. This work adds an additional capability, *i.e*. molecular computation, with a modified cTAM model, termed the *Boolean Circuit Tile Assembly Model (bcTAM)*.

**Definition 1** (bcTAM circuit). A bcTAM circuit is a tuple Ψ = (*N*, *E*, *C*, *g*, ∂*N*) where *N* and *E* represent electrical nodes and edges of a circuit respectively. Thus, the circuit is analogous to a graph (*N*, *E*). *C* is a set of circuit components required to build the tile types, and *g* is the glue set necessary for attachment among input and output nodes. ∂*N* = *N*_*in*_ ∪ *N*_*out*_ consists of input nodes and output nodes of the circuit at which glues bind tiles together. Multiple pairs of glues are possible in order to connect an output to multiple inputs, for example.

**Definition 2** (Boolean Circuit Tile Assembly Model). A Boolean cTAM assembler is a tuple C=(Γ,S,G,τ,ν,ζ), where Γ is a finite set of circuit tile types built with basic electrical circuit components, *S* ⊆ Γ is a set of seed tile types that are the starting point for the growth of an assembly, *G* ⊆ Γ is a set of gate tile types that is capable of computing Boolean logic functions, $\tau \in R_{+}$ is the threshold voltage, the parameter to determine the eligibility of further attachment, $\nu \in R_{+}$ is the node potential, *i.e*. electric potential energy at the node relative to the ground node of the circuit, and ζ maps input nodes to output nodes according to the glue rules, *i.e*. ζ: Γ(*N*_*in*_) × Γ(*N*_*out*_) → {0, 1}.

### Description of the assembly process

**Fig 1:**
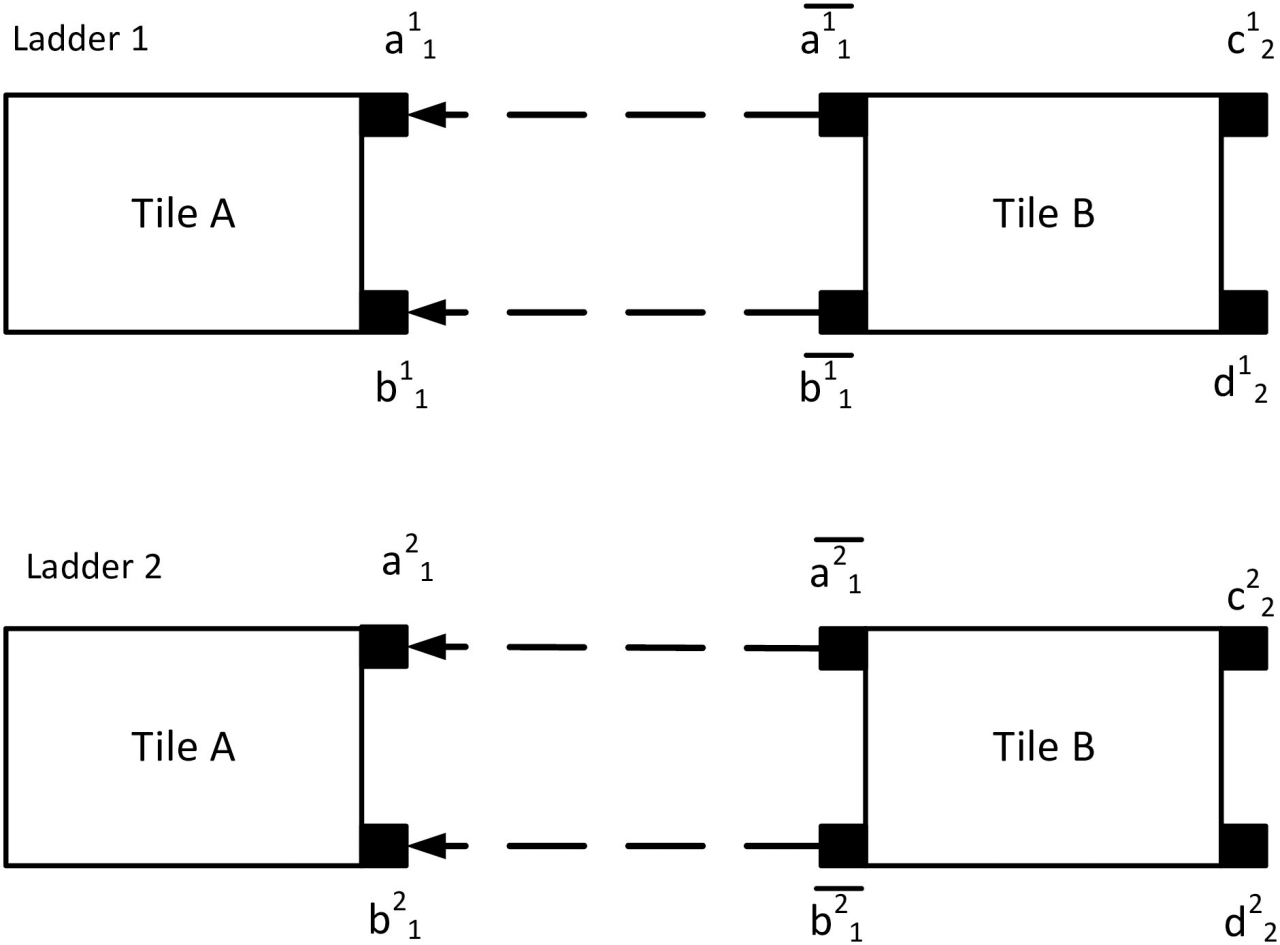
Matching glue rules. An example represents the matching glue rules. Here, the assembly system has four tiles of two tile types(tile A and tile B). The first tile A has glues $\left\{a_{1}^{1},b_{1}^{1}\right\}$ and the second tile A has glues $\left\{a_{1}^{2},b_{1}^{2}\right\}$ at the output nodes. For the tile B, one tile has glues $\left\{{\bar{a}}_{1}^{1},{\bar{b}}_{1}^{1}\right\}$, and the other tile has glues $\left\{{\bar{a}}_{1}^{2},{\bar{b}}_{1}^{2}\right\}$ at the input nodes. According to the glue rules, a stable attachment will occur between the glue pairs $\left\{a_{1}^{1}-{\bar{a}}_{1}^{1}\right\}$, $\left\{b_{1}^{1}-{\bar{b}}_{1}^{1}\right\}$, $\left\{a_{1}^{2}-{\bar{a}}_{1}^{2}\right\}$, and $\left\{b_{1}^{2}-{\bar{b}}_{1}^{2}\right\}$ if Δ*ν* ≥ *τ*, shown with dotted arrow in the figure.

An assembly describes a complete electrical circuit. It starts growing from the seed tile, and growth continues by attaching tiles based on the glue rules and a predefined threshold voltage. If the differential voltage across a node pair is greater than or equal to the threshold *τ*, the glues of the nodes are activated. The potential difference between two nodes (*p*, *q*) will be denoted as $V_{\left(p,q\right)}^{\gamma }\left(k\right)$, where the first node *p* ∈ *N* in an edge refers to the more positive potential, *γ* ∈ Γ is the tile type, and the index *k* ∈ {1, …, *n*}, denotes a specific tile in the ladder assembly of size *n*, as well as timestep. Inspired by the DNA tile assembly, the attachment rules of the cTAM is based on DNA Watson-Crick complementary oligonucleotides, where a glue matches with its complement. Each tile of the bcTAM tileset has a particular set of glues, denoted as $g_{k}^{m}$ where *g* indicates the glue type, *m* indicates the assembly number, and *k* denotes the timestep. For example, for the first step of the first ladder will have glues $g_{1}^{1}$ where *g* = *a*, *b*, …. A stable attachment may occur if the potential difference between the nodes (either input or output) is greater than the threshold voltage, and the tiles have complementary glues, *i.e*.*g* attaches to $\bar{g}$. Fig 1 shows an example of two ladders with two tile types and four tiles. Tile A, the seed tile, has two glues at the output nodes and tile B has two glues at input nodes and two glues across the output nodes. For the tile A of ladder 1, *m* = 1, *k* = 1, for the tile B of ladder 1, *m* = 1, *k* = 2, for the tile A of ladder 2, *m* = 2, *k* = 1, and for the tile B of ladder 2, *m* = 2, *k* = 2. Therefore, tile A of ladder 1 has an output node pair with glues, $a_{1}^{1}$ and $b_{1}^{1}$ that matches with tile B having glues ${\bar{a}}_{1}^{1}$ and ${\bar{b}}_{1}^{1}$ on its input node pair. A tile can attach to a growing ladder if the voltage drop across the output nodes of the ladder is greater than or equal to the threshold voltage. Here, tile A and tile B of the corresponding ladder will attach if the potential across the input or output nodes ≥ *τ*. An attachment requires four complementary glues. Since each rung of the ladder is instrumented with a gate, the required number of glues is (*n* − 1) * 4 + 2, where *n* is the length of the assembly. For this example case, the connection occurs between $\left\{a_{1}^{1}-{\bar{a}}_{1}^{1}\right\}$, $\left\{b_{1}^{1}-{\bar{b}}_{1}^{1}\right\}$, $\left\{a_{1}^{2}-{\bar{a}}_{1}^{2}\right\}$, and $\left\{b_{1}^{2}-{\bar{b}}_{1}^{2}\right\}$, shown in the example case of Fig 1. Also, tile B has glues $\left\{c_{1}^{2},d_{1}^{2}\right\}$ for ladder 1, and $\left\{c_{2}^{2},d_{2}^{2}\right\}$ for ladder 2. As *n* = 2, (2 − 1) * 4 + 2 = 6 glues are required for each of the ladders. Both of the ladders are two tile assembly, making the total number of required glues 12, as shown in Fig 1.

**Definition 3** (Terminal Circuit). A *terminal assembly* is a stable configuration in which no further attachment is possible. A circuit tile assembly model represents a dynamic circuit configuration in which growth continues based on the threshold voltage and matching glues criteria. When growth has stopped, the final circuit configuration is termed as *terminal circuit*. The number of tiles in a terminal assembly is denoted by *n*.

### Logic gates and their truth tables

In digital logic design, the most common logic gates are AND, OR, NOT, NAND, and NOR, and we can build any logic circuitry with these gates. Table 1 shows the truth table for the logic gates of AND, OR, NOR, and NAND with two inputs, and Table 2 shows the truth table of a single input NOT gate. This work aims to build a tile assembly model that has the functionalities of these five gates. The bcTAM system has one seed tile, one circuit tile, and a set of gate tiles consisting of five tile types. Each gate tile computes one particular Boolean function among the set AND, OR, NOT, NAND, NOR. Another common logic gate XOR is excluded here, as the Boolean expression of an XOR output is $\bar{A}B+A\bar{B}$, which is a combination of AND, OR, NOT gate. These five gates are enough to represent any Boolean expression, and hence, this work focuses on implementing them.

**Table 1:** Truth table of logic gates (OR, AND, NOR, NAND).

Inputs		Outputs			
Input 1	Input 2	OR Output	AND Output	NOR Output	NAND Output
0	0	0	0	1	1
0	1	1	0	0	1
1	0	1	0	0	1
1	1	1	1	0	0

**Table 2:** Truth table of NOT gate.

Input	Output
0	1
1	0

### Description of tiles

#### Seed tile

**Fig 2:**
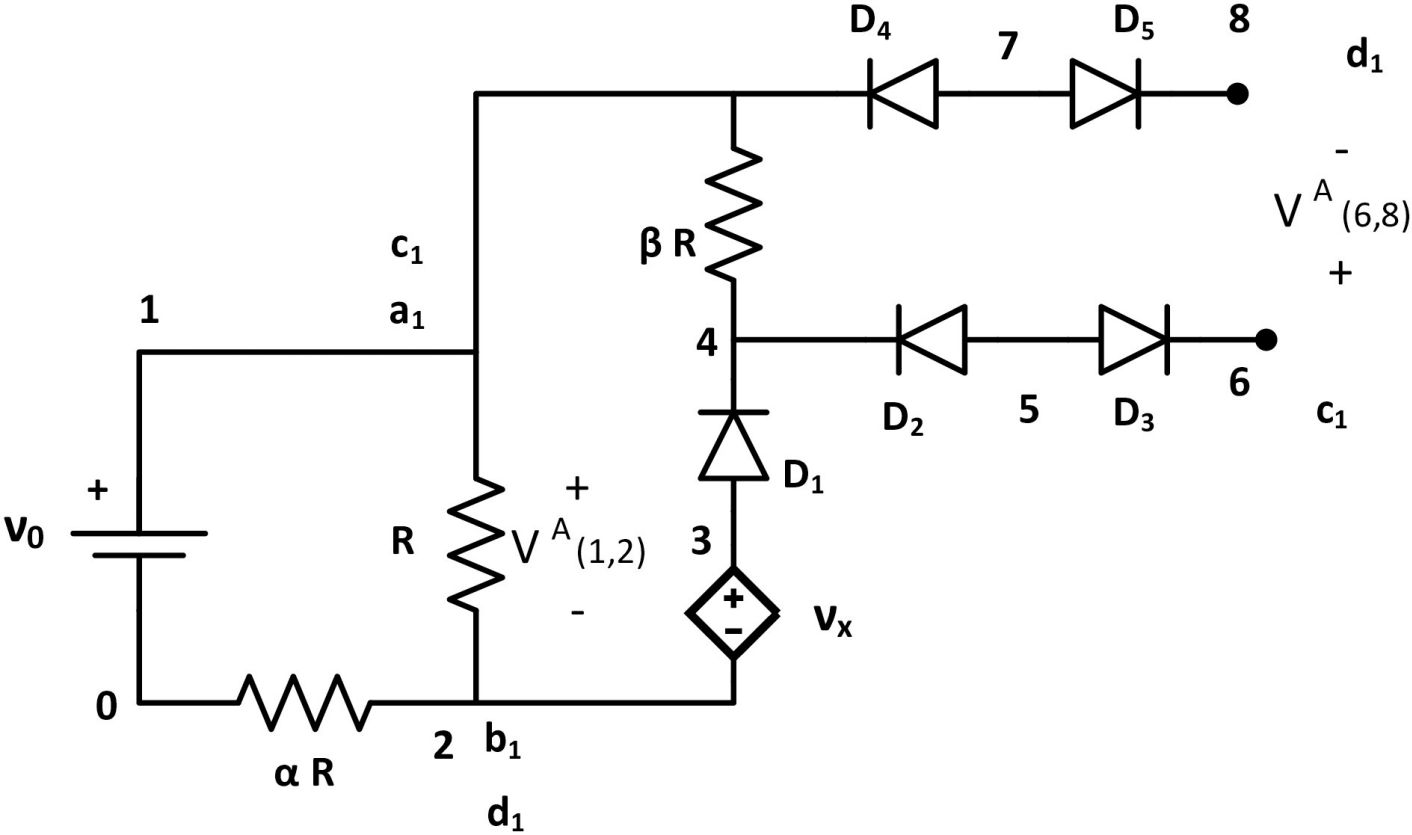
Seed tile A. Tile A (Seed Tile) for the bcTAM consisting with one DC voltage source *ν*_0_, one dependent voltage source $V_{x}=\frac{2\tau \nu_{0}}{\nu_{0}}$, five ideal diodes *D*_1_, *D*_2_, *D*_3_, *D*_4_, *D*_5_ with *τ* = 0, and three resistors (*R*, *αR*, and *βR* where *βR* is a large value resistor compared to *R* and *αR*). It has two output terminals across node {1, 2} and node {6, 8}. Node 1 has glue {*a*_1_, *c*_1_}, node 2 has glue {*b*_1_, *d*_1_}, node 6 has glue *c*_1_, and node 8 has glue *d*_1_.

Seed tile (Tile A) of bcTAM consists of two loops, where the first loop is built with one voltage source *ν*_0_ (at node {1, 0}), two resistors *R* (at node {1, 2}), and *αR* (at node {2, 0}) connected as a series circuit (Fig 2). The second loop has one large value resistor *βR* (at node {1, 4}), one dependent voltage source $V_{x}=\frac{\nu_{0}2\tau }{\nu_{0}}$ (at node {3, 2}), and one ideal diode *D*_1_ (at node {3, 4}) with threshold voltage *τ* = 0. The dependent source *V*_*x*_ equals to DC voltage source of 2*τ* if connected with *ν*_0_; otherwise it is not activated. When the dependent source is not connected to the DC voltage source, it doesnot provide any voltage supply to the second loop of the seed tile, and hence glues are not activated which is the desired condition. Four ideal diodes with zero threshold voltage are connected across the *βR* resistor: *D*_2_ at node {5, 4}, *D*_3_ at node {5, 6}, *D*_4_ at node {7, 1}, and *D*_5_ at node {7, 8}. This diode bridge prevents current flow from the next tile to the seed tile, *i.e*. they act as an isolator between two adjoining tiles.

The tile has two pairs of output nodes at {1, 2} and {6, 8} with glues *g*(1) = {*a*_1_, *c*_1_}, *g*(2) = {*b*_1_, *d*_1_}, *g*(6) = {*c*_1_}, and *g*(8) = {*d*_1_}. The first loop of seed tile A acts as a voltage divider circuit where *ν*_0_ is divided between the resistors *R* and *αR*. The second loop has a dependent voltage source that provides a 2*τ* DC voltage source if activated. According to Kirchhoff’s Voltage Law (KVL), the algebraic sum of the voltage around a closed loop of a circuit must be zero. Using KVL along nodes {1, 2, 3, 4, 1} at tile A:

$$\begin{array}{cc} & V_{x}-V_{D_{1}}-V_{\left(4,1\right)}^{A}\left(1\right)-V_{\left(1,2\right)}^{A}\left(1\right)=0,\\ & 2\tau -0-V_{\left(4,1\right)}^{A}\left(1\right)-V_{\left(1,2\right)}^{A}\left(1\right)=0,\\ & V_{\left(4,1\right)}^{A}\left(1\right)=2\tau -V_{\left(1,2\right)}^{A}\left(1\right). \end{array}$$ (1)

If $V_{\left(1,2\right)}^{A}\left(1\right)>\tau$, then $V_{\left(4,1\right)}^{A}\left(1\right)<\tau$ and if $V_{\left(1,2\right)}^{A}\left(1\right)<\tau$, then $V_{\left(4,1\right)}^{A}\left(1\right)>\tau$. From Eq 1, $V_{\left(1,2\right)}^{A}=\tau$, $V_{\left(4,1\right)}^{A}=\tau$ creates an unwanted condition of activation for both outputs $V_{\left(1,2\right)}^{A}$ and $V_{\left(6,8\right)}^{A}$. This condition can be avoided by choosing appropriate values of the input parameters (*ν*_0_, *τ*, *α*) such that the tip voltage goes from V_(1,2)_(*n* − 1) > 2*τ* for a ladder of length (*n* − 1) to V_(1,2)_(*n*) < *τ* for a ladder of length *n*, at which point the assembly terminates. To prove that there exists values of the input parameters that will reduce the tip voltage from > 2*τ* to <*τ* with the addition of a single tile, the values of the voltage will be bound, and the existence of a gap between the bound voltages shows that the condition is possible to achieve. For a ladder of length (*n* − 1), the desired condition is that V_(1,2)_(*n* − 1) > 2*τ*. Since *R* is greater than the equivalent resistance for a given length ladder, a voltage divider between *R* and *αR* is used to upper bound the voltage,

$$\begin{array}{c} 2\tau <V_{n-1}<{\left(\frac{1}{1+\alpha }\right)}^{n-1}\nu_{0}. \end{array}$$ (2)

Solving for *n*, produces

$$\begin{array}{c} 1+\frac{\text{log}(2\tau /\nu_{0})}{\text{log}(\frac{1}{1+\alpha })}<n. \end{array}$$ (3)

For a ladder of length *n*, the equivalent resistance for an infinite length ladder, $R_{eq}^{\infty }$, is used in the bound since it is less than the actual equivalent resistance. Its value

$$\begin{array}{c} R_{eq}^{\infty }=R[\frac{-\alpha +\sqrt{\alpha^{2}+4\alpha }}{2}]=R\chi . \end{array}$$ (4)

was derived in [9]. Therefore, the bound on the tip voltage for a ladder of length *n* is

$$\begin{array}{c} {\left[\frac{R_{eq}^{\infty }}{R_{eq}^{\infty }+\alpha R}\right]}^{n}\nu_{0}<V_{n}<\tau . \end{array}$$ (5)

Solving for *n* results in

$$\begin{array}{c} n<\frac{\text{log}(\tau /\nu_{0})}{\text{log}(\frac{\chi }{\chi +\alpha })}. \end{array}$$ (6)

Combining Eqs 3 and 6, requiring that Eq 6 be at least one tile larger than Eq 3, and setting *α* = 1, produces

$$\begin{array}{c} \frac{\text{log}(2\tau /\nu_{0})}{\text{log}(1/2)}<\frac{\text{log}(\tau /\nu_{0})}{\text{log}(\frac{1}{1+\phi })}, \end{array}$$ (7)

where *ϕ* is the golden ratio [8]. Solving gives

$$\begin{array}{c} 0.0839\nu_{0}<\tau , \end{array}$$ (8)

which can be satisfied for any *ν*_0_ by an appropriate choice of *τ*, proving that the condition of the tip voltage identically equal to *τ* can be avoided. Therefore, in this work, we will consider Logic 1 = HIGH (> *τ*), Logic 0 = LOW (< *τ*) and exclude the condition of tip potential is exactly equal to *τ*.

Now, when $V_{\left(4,1\right)}^{A}\left(1\right)>\tau$, the output nodes $V_{\left(6,8\right)}^{A}\left(1\right)>\tau$ due to the open loop condition at node {6, 8}. So, before any attachment, the glues of the output nodes {6, 8} are activated and ready to attach with other tiles of matching glues. However, after the attachment, it will contribute LOW (< *τ*) potential for the next tile as the diode bridge acts as an open circuit. To sum up, when the growth is continuing, output {1, 2} activates, and it provides HIGH (> *τ*) potential to attach a tile to these nodes. In contrast, when $V_{\left(1,2\right)}^{A}<\tau$, $V_{\left(6,8\right)}^{A}>\tau$, output {6, 8} activates, and it provides LOW (< *τ*) potential to the next tile attached to these nodes.

#### Circuit tile

**Fig 3:**
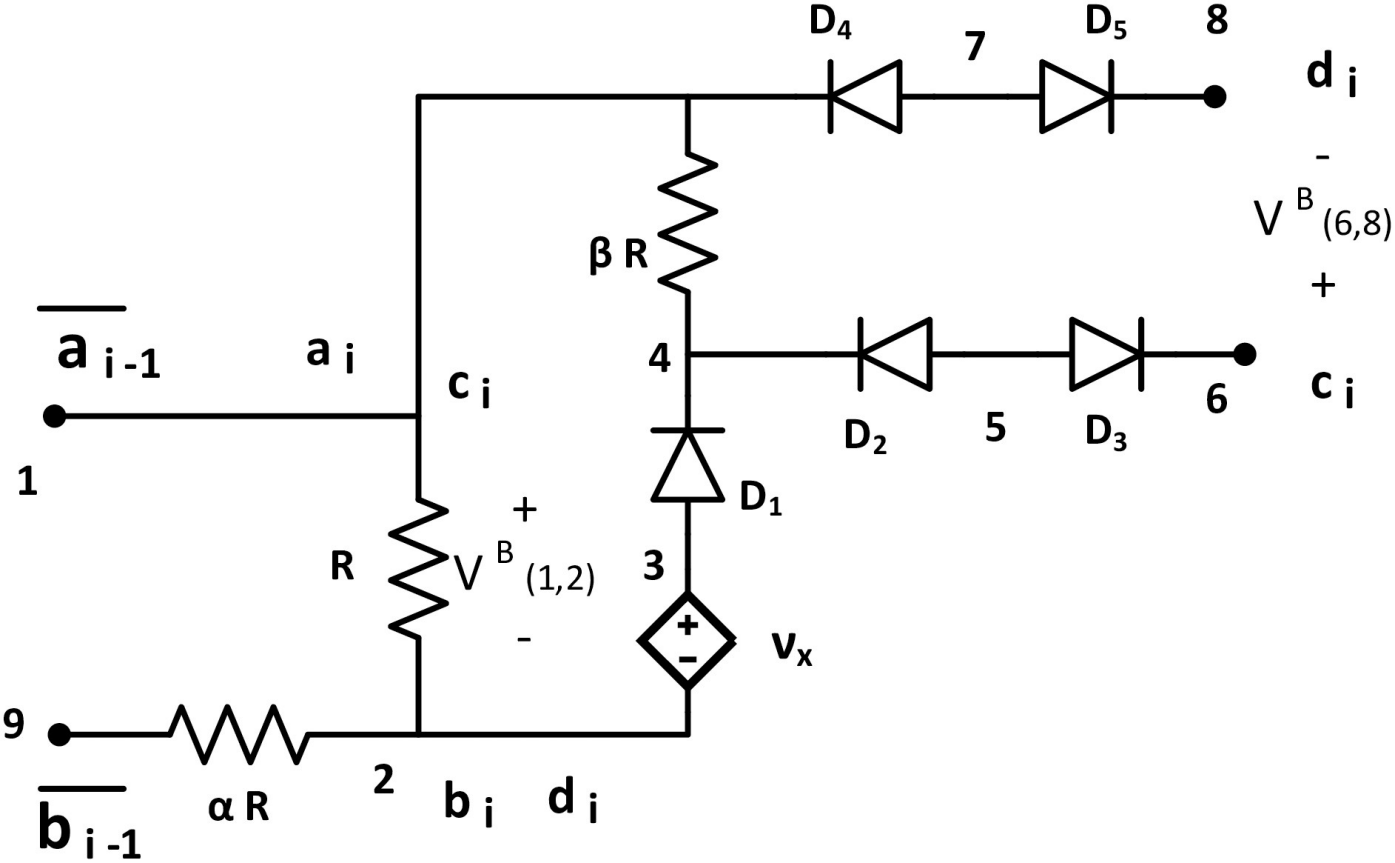
Circuit tile B. Tile B (Circuit Tile) consists with three resistors (*R*, *αR*, and *βR*), one dependent voltage source $V_{x}=\frac{2\tau \nu_{0}}{\nu_{0}}$, five ideal diodes *D*_1_, *D*_2_, *D*_3_, *D*_4_, *D*_5_ with *τ* = 0. It has one input node terminal at node {1, 9} and two output node terminals at node {1, 2} and node {6, 8}. Glues: $g\left(1\right)=\left\{a_{i},c_{i},{\bar{a}}_{i-1}\right\}$, *g*(2) = {*b*_*i*_, *d*_*i*_}, *g*(6) = *c*_*i*_, *g*(8) = *d*_*i*_, and $g\left(9\right)={\bar{b}}_{i-1}$, where *i* = 2, 3, ….

Circuit tile (Tile B) has the same circuit configuration as the seed tile except for the supply voltage (Fig 3). It has one pair of input nodes at {1, 9} and two pairs of output nodes {1, 2} and {6, 8} same as the seed tile. It has glues: $g\left(1\right)=\left\{a_{i},c_{i},{\bar{a}}_{i-1}\right\},g\left(2\right)=\left\{b_{i},d_{i}\right\},g\left(6\right)=\left\{c_{i}\right\},g\left(8\right)=\left\{d_{i}\right\}$, and $g\left(9\right)={\bar{b}}_{i-1}$, where *i* = 2, 3, …. It provides HIGH input when $V_{\left(1,2\right)}^{B}\left(k\right)>\tau$, and provides LOW input when $V_{\left(1,2\right)}^{B}\left(k\right)<\tau$.

#### OR tile

**Fig 4:**
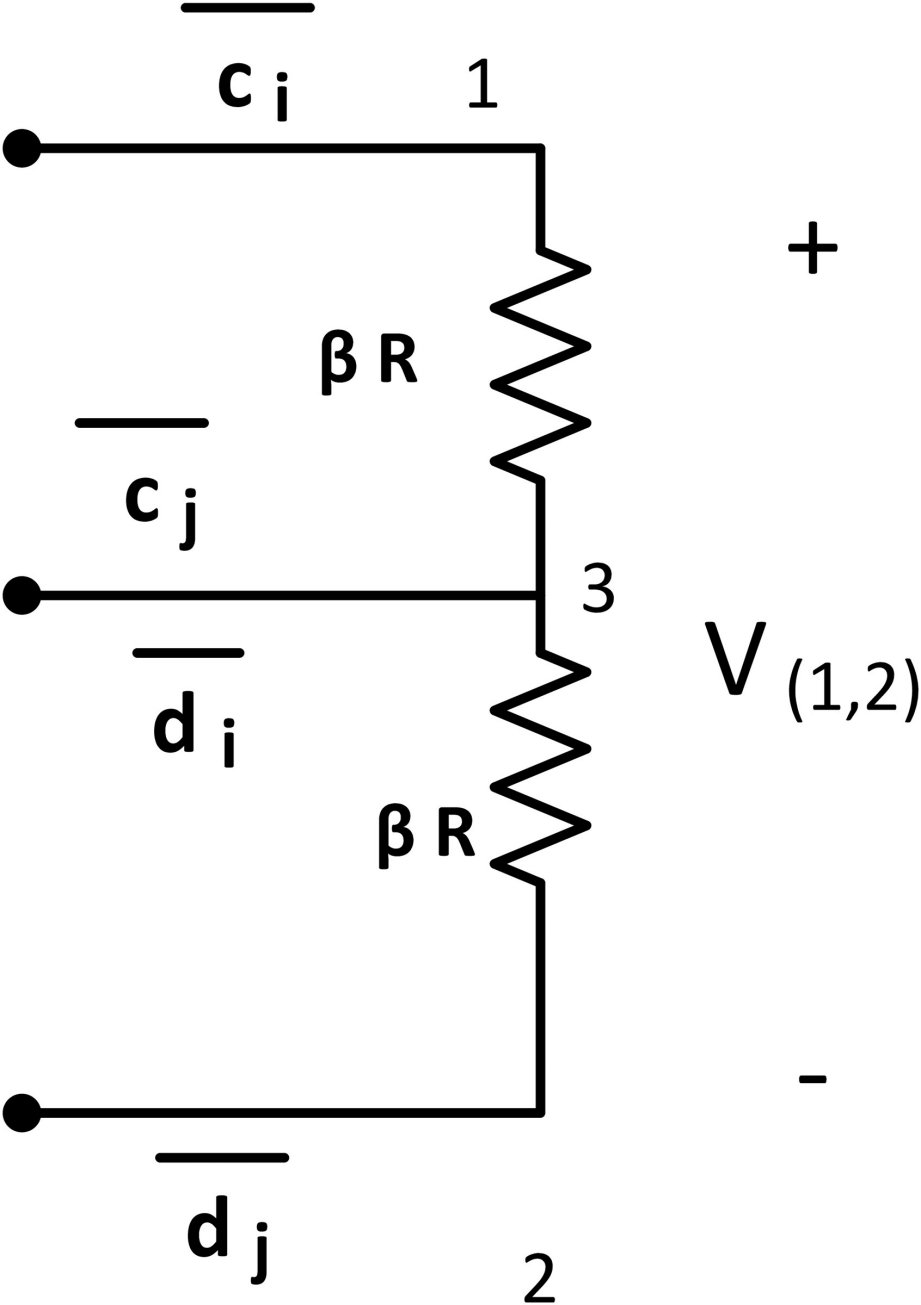
OR tile. OR tile consists of two *βR* resistors connected in series. It has two input nodes across {1, 3} and {3, 2} and one output node at {1, 2}. Glues: $g\left(1\right)=\left\{{\bar{c}}_{i}\right\},g\left(2\right)=\left\{{\bar{d}}_{j}\right\}$, and $g\left(3\right)=\left\{{\bar{c}}_{j},{\bar{d}}_{i}\right\}$, where *i* = 1, 2, … and *j* = 1, 2, … indicate the location of the assembly.

A bcTAM system has a set of gate tiles: OR tile, AND tile, NOT tile, NOR tile, and NAND tile. The gate tiles function as their name suggests, such as the OR tile works as an OR gate whose output is HIGH if any of its inputs are HIGH. The OR tile consists of two large value *βR* resistors (at node {1, 3} and {3, 2}) connected in series. It has two input node pairs across each *βR* resistors, *i.e*. at {1, 3} and {3, 2} and one output node pair at {1, 2}. It has glues: $g\left(1\right)=\left\{{\bar{c}}_{i}\right\}$, $g\left(2\right)=\left\{{\bar{d}}_{j}\right\}$, and $g\left(3\right)=\left\{{\bar{c}}_{j},{\bar{d}}_{i}\right\}$, where *i* = 1, 2, … and *j* = 1, 2, … indicate the location of the assembly (Fig 4).

For all of the two input gate tiles, *i* and *j* will indicate the location for the attachment at different assemblies. Also, the glue set will be unique for each ladder at each step. The output of OR tile is the potential across node {1, 2}, which equals to:

$$\begin{array}{cc} & V_{\left(1,3\right)}^{OR}+V_{\left(3,2\right)}^{OR}=V_{\left(1,2\right)}^{OR}. \end{array}$$ (9)

If any or both input nodes are connected with the assembly location {1, 2}, the tip potential is HIGH (> *τ*), then the output potential is also HIGH.

If any input nodes (such as {1, 3}) of the OR gate connects with the location {6, 8} of the assembly at step *k*, according to the KVL,

$$\begin{array}{cc} & V_{\left(4,1\right)}^{}\left(k\right)+V_{\left(1,7\right)}^{}\left(k\right)+V_{\left(7,8\right)}^{}\left(k\right)+V_{\left(1,3\right)}^{OR}\left(k\right)+V_{\left(6,5\right)}^{}\left(k\right)+V_{\left(5,4\right)}^{}\left(k\right)=0. \end{array}$$ (10)

As the diode *D*_2_ and *D*_4_ are reverse biased, no current can flow in this loop, and $V_{\left(1,3\right)}^{OR}\left(k\right)=0$, indicating LOW potential. From the Eq 9, if any of the inputs or both inputs ($V_{\left(1,3\right)}^{OR}$ or $V_{\left(3,2\right)}^{OR}$) are HIGH, output $V_{\left(1,2\right)}^{OR}$ is HIGH (> *τ*). If both input is LOW (*i.e*. Zero), then the output $V_{\left(1,2\right)}^{OR}$ is LOW. Therefore, it matches with the truth table of an OR gate. The two-input OR gate can be modified for an *m* input OR gate by adding *m* number of *βR* resistors and unique glues.

#### NOT tile

**Fig 5:**
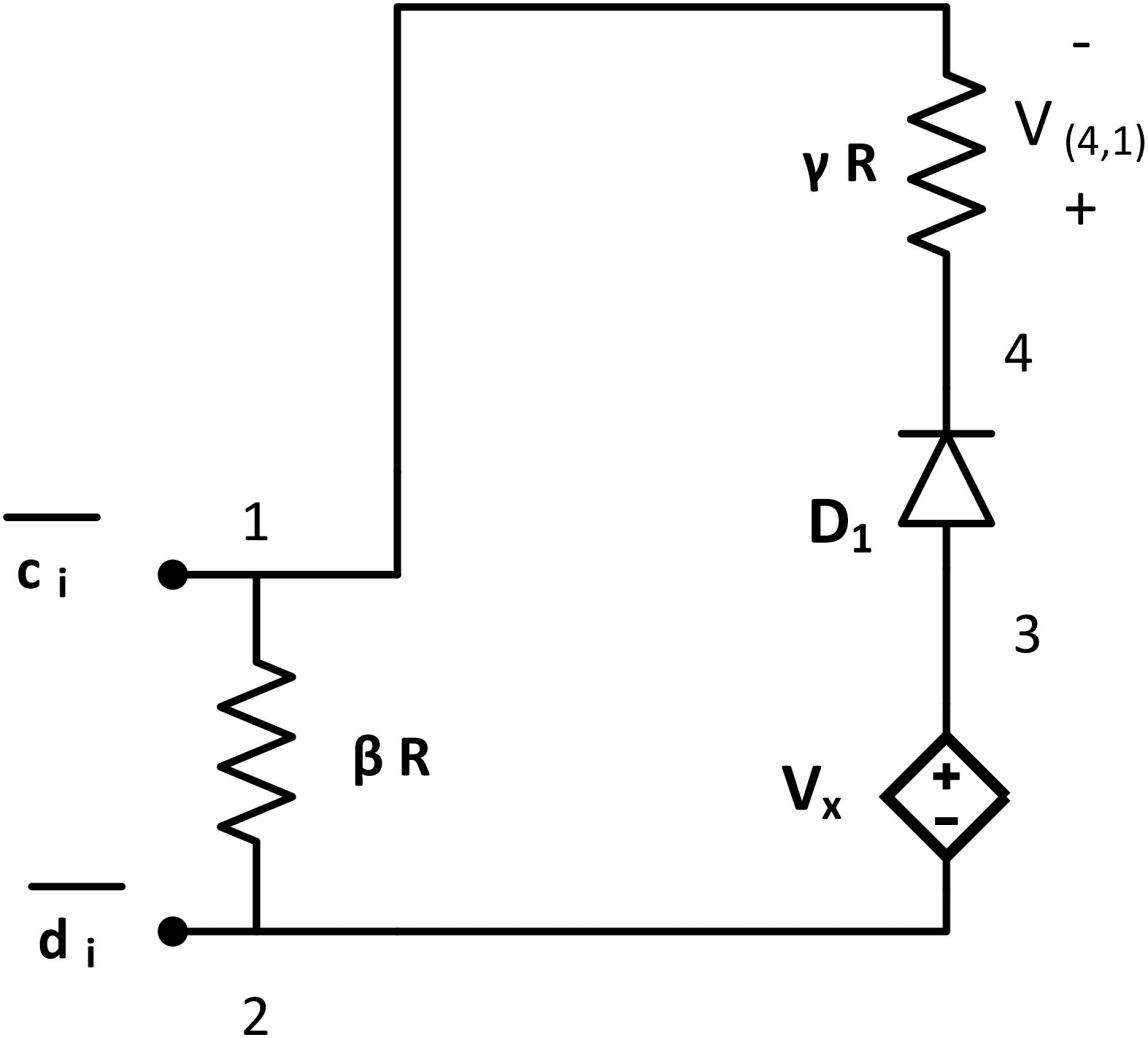
NOT tile. NOT tile consists of two large value resistors (*βR* and *γR*, where *γ* > >*β*), one ideal diode *D*_1_ with *V*_*thr*_ = 0, and one dependent voltage source $V_{x}=\frac{2\tau \nu_{0}}{\nu_{0}}$ in series connection. It has input node across node {1, 2} and output node at {4, 1}. Glues: $g\left(1\right)=\left\{{\bar{c}}_{i}\right\},g\left(2\right)=\left\{{\bar{d}}_{i}\right\}$.

The NOT tile implements logical negation of its input. It is a single loop circuit with two large value resistors (*βR* at node {1, 2} and *γR* at node {4, 1} and *γ* > >*β*), one ideal diode *D*_1_ at node {3, 4}, and one dependent voltage source of $V_{x}=\frac{\nu_{0}2\tau }{\nu_{0}}$ at {3, 2}. The threshold voltage of the diode, *V*_*thr*_ = 0. It has one input node at {1, 2} and one output node at {4, 1}. It has glues: $g\left(1\right)={\bar{c}}_{i}$ and $g\left(2\right)={\bar{d}}_{i}$ (Fig 5).

The working mechanism of the NOT tile is similar to NOT gate. Using KVL along the tile:

$$\begin{array}{cc} & V_{x}-V_{D_{1}}-V_{\left(4,1\right)}^{NOT}-V_{\left(1,2\right)}^{NOT}=0,\\ & 2\tau -0-V_{\left(4,1\right)}^{NOT}-V_{\left(1,2\right)}^{NOT}=0,\\ & V_{\left(4,1\right)}^{NOT}=2\tau -V_{\left(1,2\right)}^{NOT}. \end{array}$$ (11)

If the NOT tile has logic HIGH as input *i.e*. $V_{\left(1,2\right)}^{NOT}\left(k\right)>\tau$, using Eq 11, $V_{\left(4,1\right)}^{NOT}<\tau$. In contrast, if the tile is connected with a terminal circuit at node {6, 8}, it gets a LOW input across resistor *βR*. Then, $V_{\left(1,2\right)}^{NOT}\left(k\right)<\tau$ and from the Eq 11, $V_{\left(4,1\right)}^{NOT}>\tau$. Thus, the NOT tile’s output potential is inverted with respect to its input potential, acting like a NOT gate (Table 2).

#### AND tile

**Fig 6:**
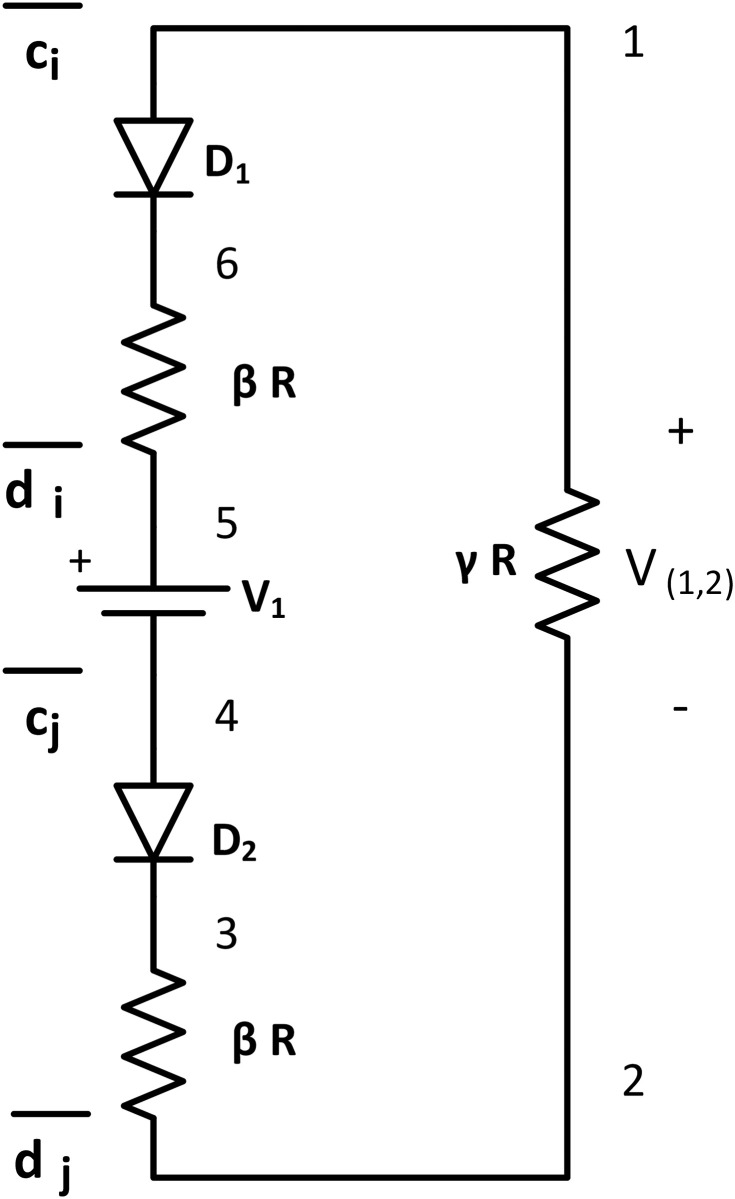
AND tile. AND tile consists of two ideal diodes *D*_1_ and *D*_2_ with *V*_*thr*_ = *τ*, two large value *βR* resistors, one large value *γR* resistor, and one DC voltage source *V*_1_ = *τ*. It has input nodes across node pair {1, 5} and {4, 2}, and output node at {1, 2}. Glues: $g\left(1\right)=\left\{{\bar{c}}_{i}\right\},g\left(2\right)=\left\{{\bar{d}}_{j}\right\},g\left(4\right)=\left\{{\bar{c}}_{j}\right\},g\left(5\right)=\left\{{\bar{d}}_{i}\right\}$.

The AND tile implements logical conjunction where a HIGH output results if all the inputs of the AND tile are HIGH. The AND tile consists of a series connection among two diodes *D*_1_, *D*_2_(Ideal diodes with threshold *τ*), one DC voltage source *V*_1_ = *τ*, and three resistors (Two *βR* resistors and one *γR* resistor where *γ* > >*β* > >*R*) (Fig 6). It has two input nodes at {1, 5} and {4, 2}. The output nodes are across *γR* resistor at node {1, 2}. It has glues: $g\left(1\right)={\bar{c}}_{i}$, $g\left(5\right)={\bar{d}}_{i}$, $g\left(4\right)={\bar{c}}_{j}$, $g\left(2\right)={\bar{d}}_{j}$.

When an AND tile is floating (not connected with the seeded assembly), both diodes are reverse-biased, and no current flows through the *γR* resistor. If any input nodes ($V_{\left(1,5\right)}^{AND}$ or $V_{\left(4,2\right)}^{AND}$) connects with the LOW potential output terminal, *i.e*. node {6, 8} of the assembly, the corresponding diode of AND tile is still in reverse bias condition, acts as an open circuit, no current flows through the tile, and hence $V_{\left(1,2\right)}^{AND}=0<\tau$. If both of the input nodes are connected with node pair {1, 2} of the growing assembly, they get HIGH potential (> *τ*). So, both diodes become forward bias, current flows through the loop {5, 6, 1, 2, 3, 4, 5}. Using KVL at the loop:

$$\begin{array}{cc} & V_{1}+V_{\left(5,1\right)}^{AND}-V_{\left(1,2\right)}^{AND}+V_{\left(2,4\right)}^{AND}=0,\\ & V_{\left(1,2\right)}^{AND}=\tau +V_{\left(5,1\right)}^{AND}+V_{\left(2,4\right)}^{AND}. \end{array}$$ (12)

If both diodes *D*_1_ and *D*_2_ are forward biased, $V_{\left(5,1\right)}^{AND}$ and $V_{\left(2,4\right)}^{AND}$ are greater than *τ*. Using Eq 12, $V_{\left(1,2\right)}^{AND}>\tau =$ HIGH output. These properties match with a two-input AND gate. Same as the OR tile, it can be modified to make it an *m* input AND gate by adding *m* number of diode-resistor pairs on the input side with new glue pairs.

#### NOR tile

**Fig 7:**
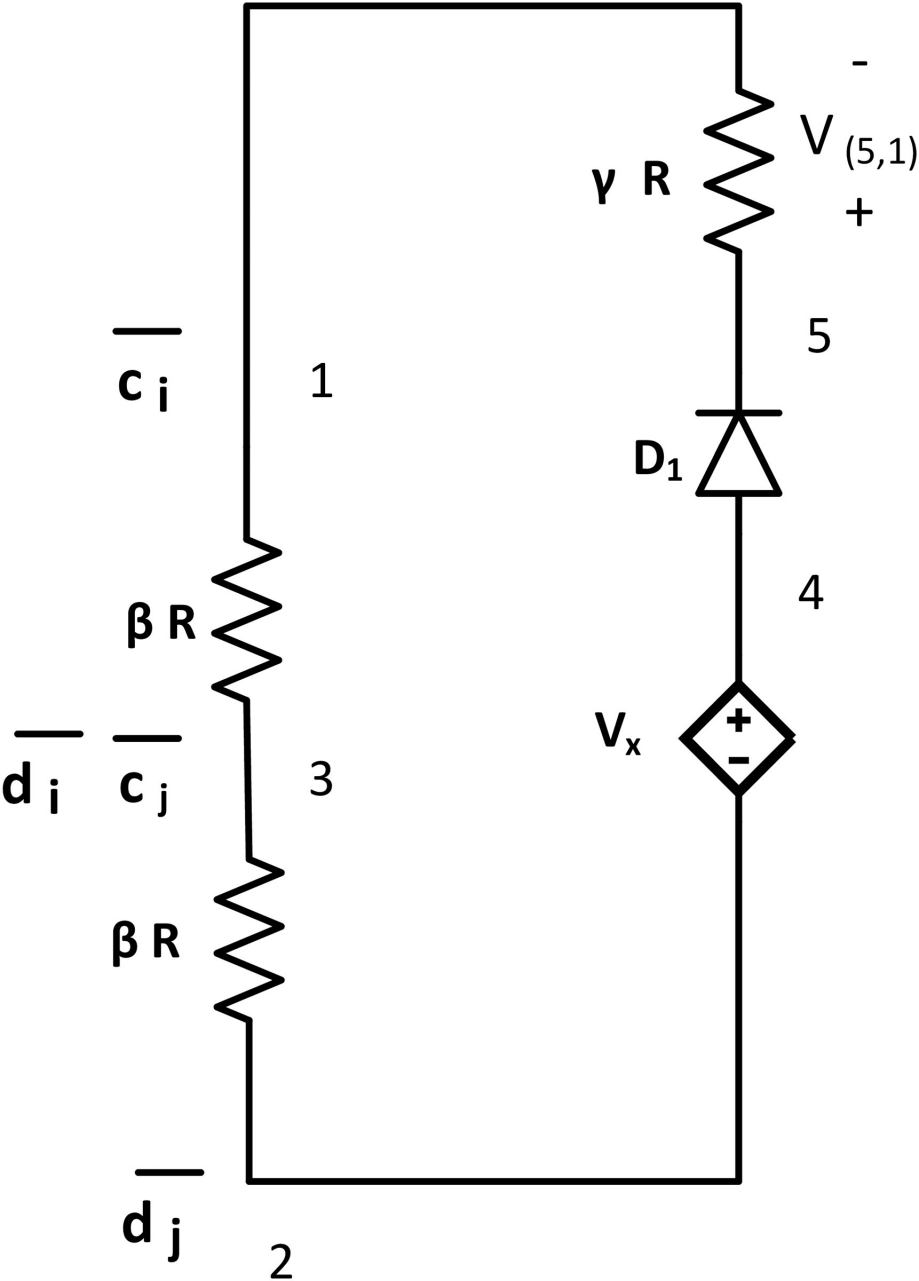
NOR tile. NOR tile has three resistors (two *βR* resistors, and *γR* resistors where *γ* > >*β* > >*R*), one ideal diode *D*_1_ with zero threshold voltage, and one dependent voltage source $V_{x}=\frac{2\tau \nu_{0}}{\nu_{0}}$. It has two input node pairs across node {1, 3} and {3, 2}. This tile has an output node at {5, 1}. Glues: $g\left(1\right)=\left\{{\bar{c}}_{i}\right\},g\left(2\right)=\left\{{\bar{d}}_{j}\right\}$, and $g\left(3\right)=\left\{{\bar{c}}_{j},{\bar{d}}_{i}\right\}$.

The NOR tile works as a NOR gate of a digital logic design, where a HIGH output results if both of the inputs are LOW. Fig 7 shows a NOR tile with *bcTAM*. It has one loop consisting of three resistors in series (*βR* resistor at node {1, 3}, *βR* resistor at {3, 2}, and *γR* resistor at node {5, 1} where *γ* > >*β*), an ideal diode *D*_1_ at node {4, 5} with *V*_*thr*_ = 0, and one dependent voltage source $V_{x}=\frac{\nu_{0}2\tau }{\nu_{0}}$ at node {4, 2}. It has two input nodes across two *βR* resistors and output nodes across *γR* resistor. The glues are:$g\left(1\right)=\left\{{\bar{c}}_{i}\right\}$, $g\left(2\right)=\left\{{\bar{d}}_{j}\right\}$, and $g\left(3\right)=\left\{{\bar{c}}_{j},{\bar{d}}_{i}\right\}$.

The NOR tile can attach to two assemblies with a complementary glues {*c*_*i*_ − *d*_*i*_} or {*c*_*j*_ − *d*_*j*_}. Applying KVL to the loop:

$$\begin{array}{cc} & V_{x}-V_{D_{1}}-V_{\left(5,1\right)}^{NOR}-V_{\left(1,3\right)}^{NOR}-V_{\left(3,2\right)}^{NOR}=0,\\ & 2\tau -0-V_{\left(5,1\right)}^{NOR}-V_{\left(1,3\right)}^{NOR}-V_{\left(3,2\right)}^{NOR}=0,\\ & V_{\left(5,1\right)}^{NOR}=2\tau -V_{\left(1,3\right)}^{NOR}-V_{\left(3,2\right)}^{NOR}. \end{array}$$ (13)

If both inputs ($V_{\left(1,3\right)}^{NOR}$ and $V_{\left(3,2\right)}^{NOR}$) are LOW, the potential is approximately zero as per our previous discussion. From Eq 13, $V_{\left(5,1\right)}^{NOR}=2\tau -0=2\tau$, which indicates HIGH output. But if both or either of the inputs are HIGH, $V_{\left(5,1\right)}^{NOR}<\tau$, indicating LOW output. So, the output is HIGH iff both inputs are LOW, and the output is LOW otherwise, representing the NOR operation.

#### NAND tile

**Fig 8:**
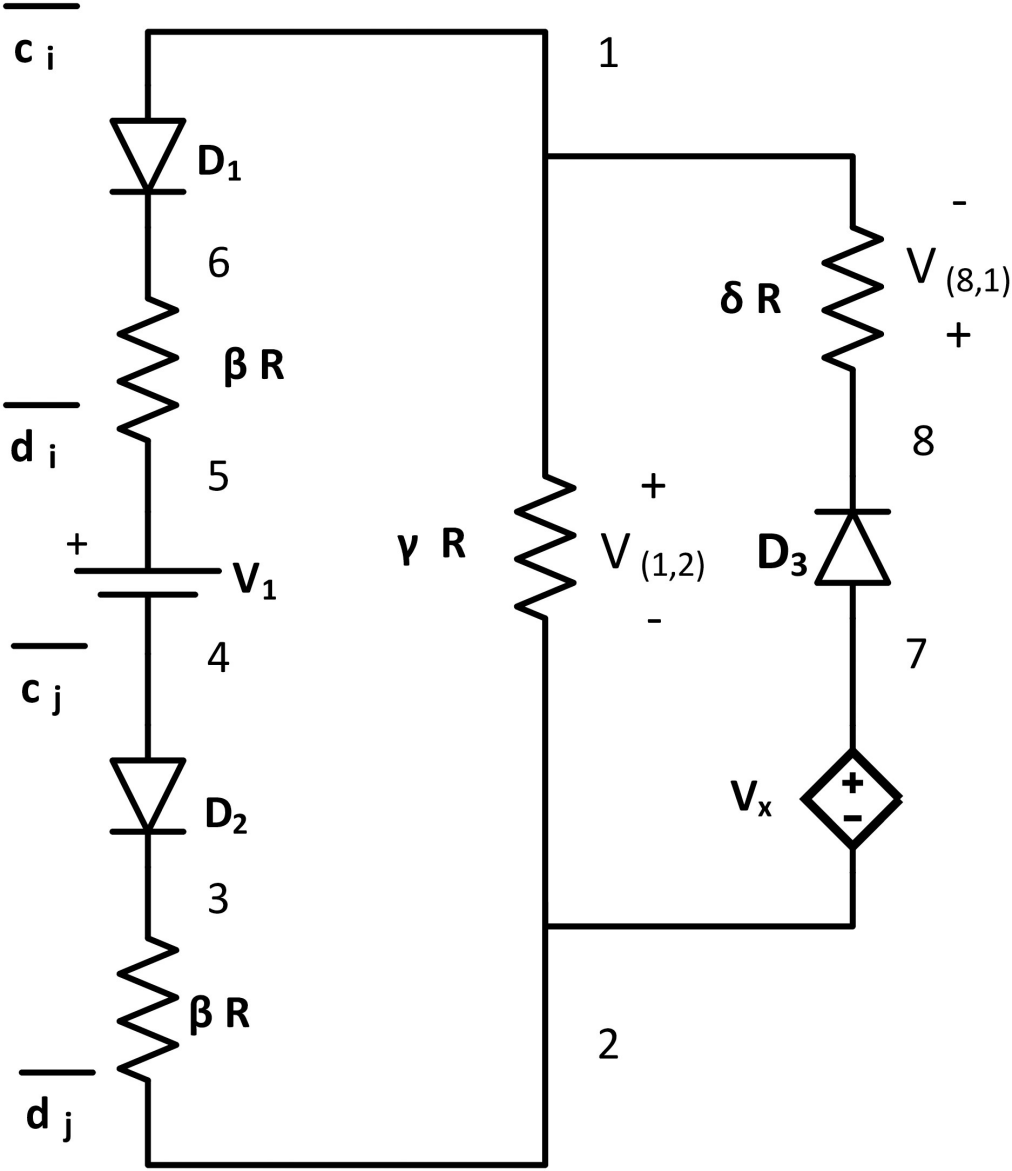
NAND tile. NAND tile consists of three ideal diodes (Diode *D*_1_, *D*_2_ have *V*_*thr*_ = *τ*, and diode *D*_3_ has *V*_*thr*_ = 0), four resistors (Two *βR* resistors, one *γR* resistor, and one *δR* resistor, where *δ* > >*γ* > >*β* > >*R*), and two voltage sources (One DC voltage source *V*_1_ = *τ*, and one dependent voltage source of $V_{x}=\frac{2\tau \nu_{0}}{\nu_{0}}$). It has two input terminals across node pairs {1, 5} and {4, 2}, and the output terminal at {8, 1}. Glues: $g\left(1\right)=\left\{{\bar{c}}_{i}\right\},g\left(2\right)=\left\{{\bar{d}}_{j}\right\},g\left(4\right)=\left\{{\bar{c}}_{j}\right\},$ and $g\left(5\right)=\left\{{\bar{d}}_{i}\right\}$.

The last tile for the logic gate set of the *bcTAM* is the NAND tile. The functionality of this tile is the same as a NAND gate, where the output is HIGH if both inputs are LOW or any one of its inputs is LOW. This tile has two loops. The first loop has one voltage source *V*_1_ = *τ* (at node {5, 4}), three resistors: Two *βR* resistors (at node {5, 6} and {2, 3}), one *γR* resistor (at node {1, 2}), two ideal diodes *D*_1_ (at node {1, 6}) and *D*_2_ (at node {4, 3}) with *V*_*thr*_ = *τ*. The second loop is similar to the NOT tile with one dependent voltage source *V*_*x*_ (at node {7, 2}), one ideal diode *D*_3_ (at node {7, 8}) with *τ* = 0, and one *δR* resistor at {8, 1}. Among the resistor values, *δ* > >*γ* > >*β* > >*R*. It has input nodes across node pair {1, 5} and {4, 2}, and output node at {8, 1} (Fig 8). The glues are: $g\left(1\right)=\left\{{\bar{c}}_{i}\right\}$, $g\left(2\right)=\left\{{\bar{d}}_{j}\right\}$, $g\left(4\right)=\left\{{\bar{c}}_{j}\right\}$, $g\left(5\right)=\left\{{\bar{d}}_{i}\right\}$.

The tile acts as a two-input NAND gate for the Boolean circuit tile assembly model. The first loop is the same as AND tile, and the second loop is the same as NOT Tile. Using KVL for the second loop:

$$\begin{array}{c} V_{\left(8,1\right)}^{NAND}=2\tau -V_{\left(1,2\right)}^{NAND}. \end{array}$$ (14)

From the working principle of AND tile, it is proven that if both of the diodes *D*_1_ and *D*_2_ are forward biased due to the HIGH input node potential, then $V_{\left(1,2\right)}^{NAND}>\tau$. From Eq 14, $V_{\left(8,1\right)}^{NAND}<\tau$. In contrast, if any or both input diodes are reverse biased due to the LOW input node potential, $V_{\left(1,2\right)}^{NAND}<\tau$ and $V_{\left(8,1\right)}^{NAND}>\tau$. Thus, all the input conditions for the NAND truth table are satisfied with the NAND tile.

### Sensing growth with bcTAM

This section will discuss an example problem and its solution using bcTAM that shows how Boolean computations determine when a set of ladders have stopped growing. The set of ladders are supplied with variable input voltages. Each tile of each ladder must be connected to a logic gate tile. To demonstrate, the case of two growing ladders is highlighted. We can design it with two seed tiles (tile A), multiple circuit tiles (tile B), and multiple NOR tiles (tile C). We annotated the tileset based on the number of seed tiles, as the number of seed tiles decides the number of ladders. As the system has two seed tiles, two ladders will grow; hence, there will be two distinct glue sets: *i* and *j*. The glues are denoted as $g_{k}^{m}$ where *g* indicates the glue types (such as *a*, *b*, *c*, *d*), *m* indicates the assembly number, and *k* denotes the timestep. For example, the first assembly (*m* = 1) will have *a* glues as $a_{1}^{1},a_{2}^{1},a_{3}^{1}$ and the second assembly will have glues $a_{1}^{2},a_{2}^{2},a_{3}^{2}$ for *k* = 1, 2, 3, respectively. The same glue notations will be used for other glues: *b*, *c*, *d*. Except for the seed tile, there is no independent voltage source in other tiles. Therefore, all output node potentials will be less than the threshold as the dependent source is not activated until it is attached to the *ν*_0_.

**Fig 9:**
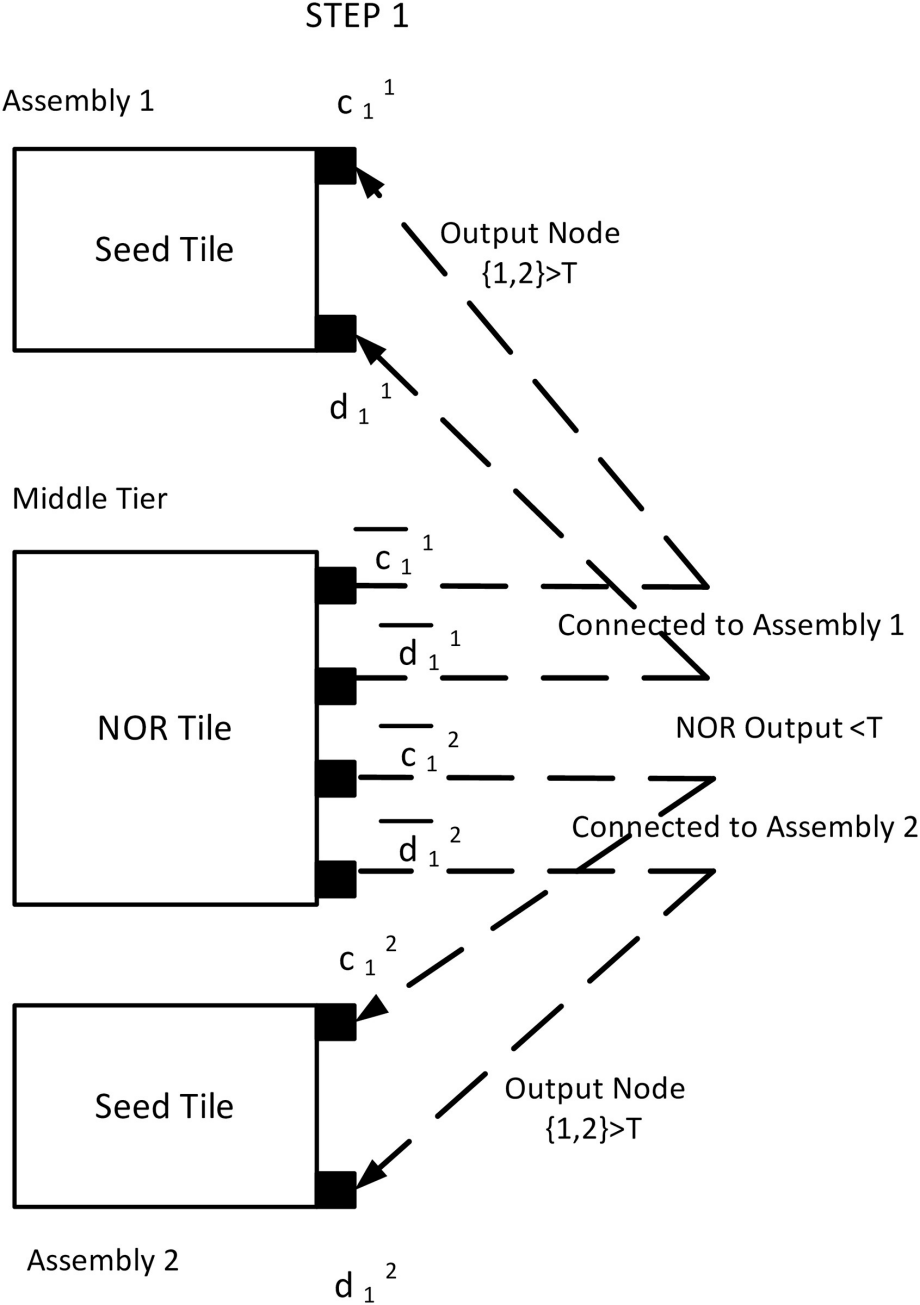
Step 1 of the example assembly. Fig. shows the step 1 for an example case with two seed tiles. Both of the assemblies have $V_{\left(1,2\right)}^{A}>\tau$. A NOR tile will attach to both of them and the NOR output is <*τ*.

The growth starts from the seed tiles and compares the tip potential to the threshold voltage. If tip potential is higher than the threshold *τ*, another tile will attach based on the glue rules. The assembly starts with the seed tile (tile A). Let us assume both tile A has source potential >*τ* as well as $V_{\left(1,2\right)}^{A}>\tau$. Also, we assume, the source potentials for the first and second assemblies are *ν*_01_ and *ν*_02_ respectively where $\nu_{0_{1}}<\nu_{0_{2}}$. For both of the assemblies, $V_{\left(1,2\right)}^{A}\left(1\right)>\tau$, it activates the attached glues *i.e*. $\left\{a_{1}^{1}-b_{1}^{1}\right\}$, $\left\{c_{1}^{1}-d_{1}^{1}\right\}$, $\left\{a_{1}^{2}-b_{1}^{2}\right\}$, and $\left\{c_{1}^{2}-d_{1}^{2}\right\}$. A circuit tile with complementary glues $\left\{{\bar{a}}_{1}^{1}-{\bar{b}}_{1}^{1}\right\}$ attaches to the first assembly at node {1–2}. Similarly, the second assembly attaches with the circuit tile having matching glues. A NOR tile (tile C) with input glues $\left\{{\bar{c}}_{1}^{1},{\bar{d}}_{1}^{1}\right\},\left\{{\bar{c}}_{1}^{2},{\bar{d}}_{1}^{2}\right\}$ also attaches to node pair {1–2}. Since $V_{\left(1,2\right)}^{C}\left(1\right)>\tau$ in NOR tile 1, $V_{\left(5,1\right)}^{C}\left(1\right)<\tau$ that indicates a LOW state (Fig 9).

**Fig 10:**
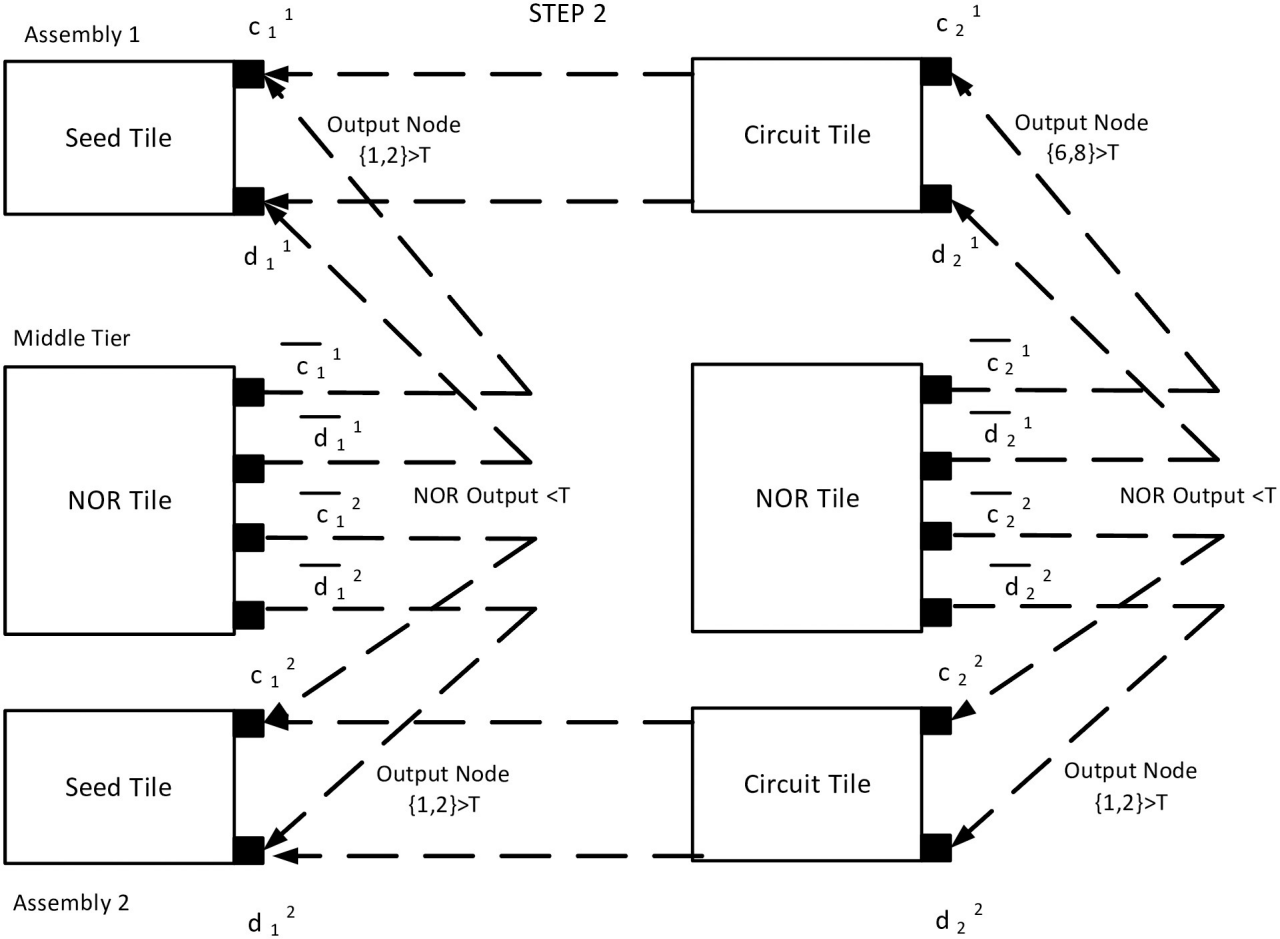
Step 2 of the example assembly. Here, the circuit tiles are connected to the assemblies. The second assembly has $V_{\left(1,2\right)}^{B}>\tau$, whereas the first assembly has $V_{\left(1,2\right)}^{B}<\tau$. Still, one input of the NOR tile is greater than the threshold and thus, NOR output is still <*τ*.

For the next step, in tile B, input potential $V_{\left(1,9\right)}^{B}\left(2\right)$ is further distributed in circuit components. Let us assume, $V_{\left(1,2\right)}^{B}\left(2\right)<\tau$ in assembly 1, and $V_{\left(1,2\right)}^{B}\left(2\right)>\tau$ in assembly 2. For the first assembly, according to the Kirchoff’s Voltage Law across the loop {1, 2, 3, 4, 1}, $V_{\left(4,1\right)}^{B}\left(2\right)=V_{\left(6,8\right)}^{B}\left(2\right)>\tau$. It activates glue $\left\{c_{2}^{1}-d_{2}^{1}\right\}$ only and a NOR tile C with glue $\left\{{\bar{c}}_{2}^{1},{\bar{d}}_{2}^{1}\right\}$ will attach to the node {6, 8}, and no further circuit tiles can attach to the assembly. But in case of the second assembly, $V_{\left(1,2\right)}^{B}\left(2\right)>\tau$ and it activates both the glues $\left\{a_{2}^{2}-b_{2}^{2}\right\}$ and $\left\{c_{2}^{2}-d_{2}^{2}\right\}$. A circuit tile and a NOR tile with complementary glues will attach to the assembly at node {1, 2}. As the tile C is still having input greater than *τ*, $V_{\left(5,1\right)}^{C}\left(2\right)<\tau$, that means LOW output (Fig 10).

**Fig 11:**
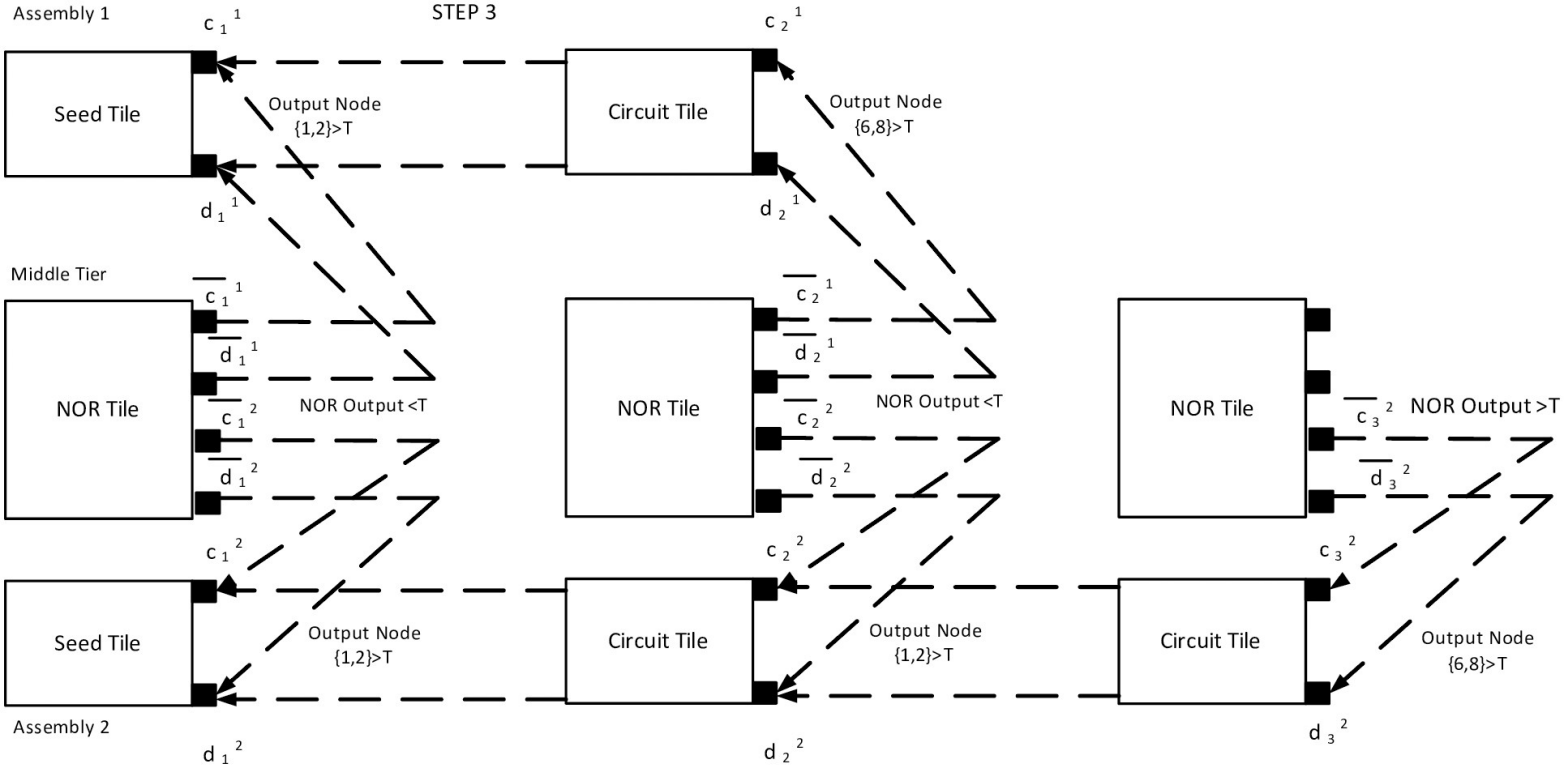
Step 3 of the example assembly. Fig. shows the third step for the example case. Both of the assemlies have $V_{\left(1,2\right)}^{B}\left(3\right)<\tau$ and the NOR output is also < *τ*.

**Fig 12:**
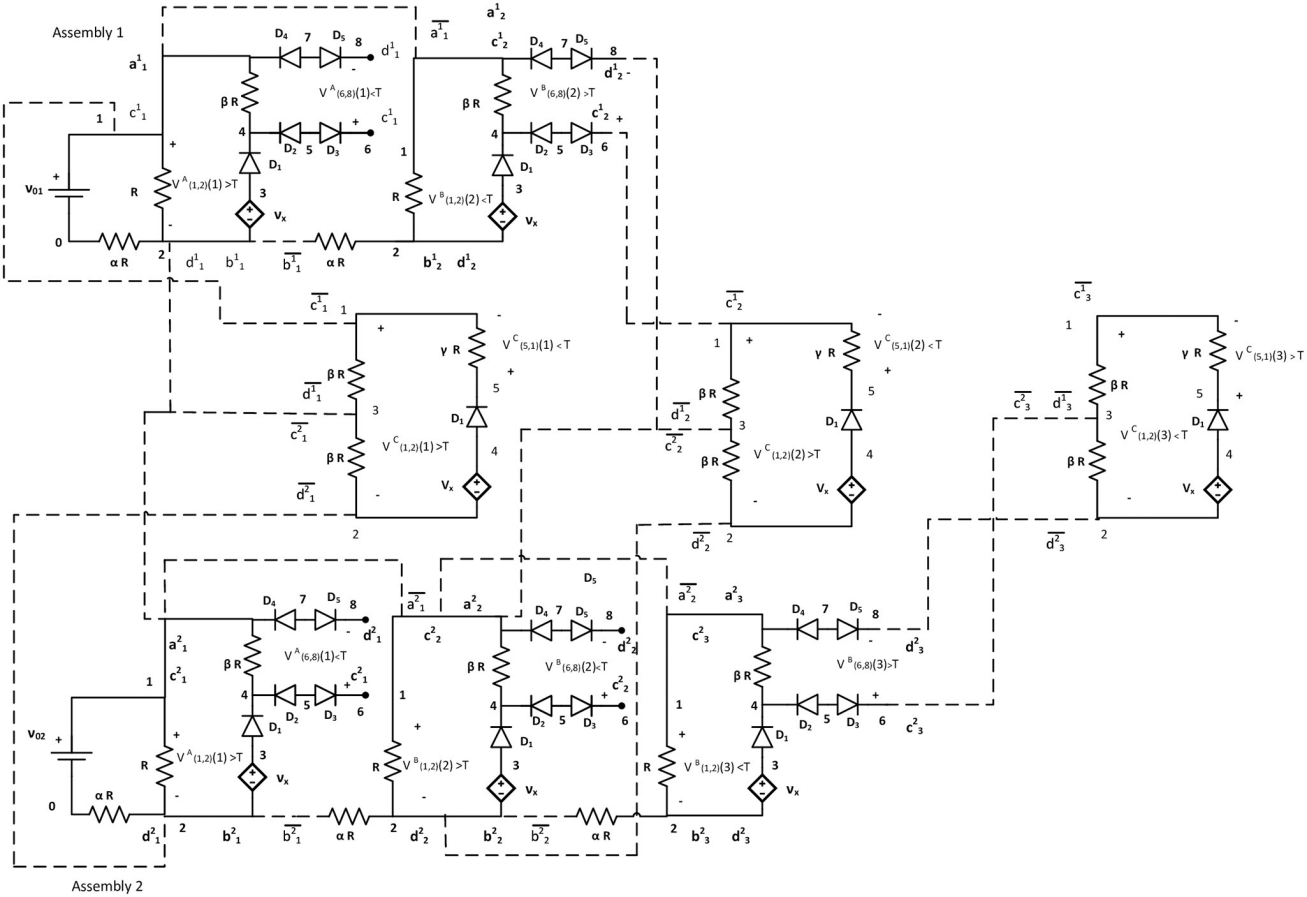
Circuit configuration of the example assembly. Fig. shows the circuit configuration of the example assembly that represents the working mechanism of a NOR gate. Here, two assemblies are growing simultaneously. The dotted lines show attachment with glues. The first assembly has a length of two, and the second assembly has a length of three. As long as both of the assemblies, or any one of them is growing, the output potential of NOR tile (middle tier), $V_{\left(5,1\right)}^{C}\left(k\right)<\tau =LOW$. When both assemblies are terminals, $V_{\left(5,1\right)}^{C}\left(t\right)>\tau =HIGH$.

In the third timestep, we assume, second assembly also has less than *τ* tip potential *i.e*. $V_{\left(1,2\right)}^{B}\left(3\right)<\tau$ and $V_{\left(6,8\right)}^{B}\left(3\right)>\tau$. It activates only $\left\{c_{3}^{2}-d_{3}^{2}\right\}$ glues. Hence, only a NOR tile attaches with glues $\left\{{\bar{c}}_{3}^{2},{\bar{d}}_{3}^{2}\right\}$. As per the mechanism described in the earlier section, the input potential of tile C is LOW. In tile C, $V_{\left(1,2\right)}^{C}\left(3\right)<\tau$ resulting in $V_{\left(5,1\right)}^{C}\left(3\right)>\tau$, a HIGH state of output (Fig 11). Thus, the output terminal of NOR tile, $V_{\left(5,1\right)}^{C}\left(k\right)$ is HIGH(>*τ*) iff both assemblies are in a terminal configuration and acts as an indicator of the moment when the system has no growing assembly. Figs 9–11 show the block diagram representaion of the step by step assembly process and Fig 12 shows the terminal configuration of the example with circuit configuration.

## bcTAM computation of SAT

The bcTAM, a model of biological growth in which electric potential is the driving force, is capable of implementing a complete set of Boolean gates with which all Boolean functions can be realized. The example of the last section can be generalized to ask more complicated Boolean questions about the growth of a set of ladders. The input voltages from the seeds will have different values and represent different signals that drive growth. In general, input potentials to the logic gates can come from the seeds or any other output nodes of the bcTAM circuit, *i.e*. rungs of the ladder or outputs of gates. These potentials and their negations represent potentially complex signals that promote or inhibit growth. The bcTAM gates are then capable of computing Boolean functions related to growth on that set of potentials.

**Definition 4** (Satisfiable bcTAM Circuit). A satisfiable bcTAM circuit (Definition 1) is a circuit of seeds, ladders, and gates produced by a bcTAM growth process that has a single TRUE output (tip potential greater than the threshold).

The computational complexity and power of the bcTAM to make Boolean-based decisions about complex problems related to the growth of a set of ladders is motivated by the following decision problem:

**Definition 5** (bcTAM SATISFIABILITY). INSTANCE: A bcTAM (Definition 2) in which the set of seed tiles *S* = {*s*_1_, *s*_2_, …, *s*_*m*_} are assigned arbitrary input voltages $\nu =\{\nu_{0}^{1},\nu_{0}^{2},\ldots ,\nu_{0}^{m}\}$.

QUESTION: Is there an assignment of input voltages *ν* to *S* such that the cTAM assembles a satisfiable circuit, *i.e*. one with a single output on a specific gate whose value is TRUE?

**Theorem 0.1**. bcTAM Satisfiability is NP-Complete.

*Proof*. A non-deterministic algorithm for bcTAM Satisfiability would guess the values of the seed voltages *ν* and determine whether the circuit was satisfiable in a polynomial time determined by the number of ladder tiles and gates, and thus, bcTAM Satisfiability is in NP. The NP-Hardness of bcTAM Satisfiability will be proven by reduction from the known NP-complete decision problem Satisfiability(SAT) [22, 23], which consists of a boolean formula composed of clauses with AND, OR, and NOT functions in conjunctive normal form.

**Definition 6** (Satisfiability (SAT) [23]). Instance: A set of U variables and a collection of clauses C over U.

Question: Is there a satisfying truth assignment for C?

The basic idea of the proof is to construct the Boolean formula in the clauses C in a bcTAM. Each variable in *U* is associated with one of the seed tiles; thus, |*U*| = |*S*|. Values of the variables *U* correspond to seed voltages such that a FALSE corresponds to a voltage less than the threshold and a TRUE is a voltage greater than or equal to the threshold. These voltage values can also be chosen such that the output of the seed tiles reflects the correct values for *U*. Each disjunctive (OR) clause in *C* is represented by a bcTAM OR gate. The glue rules implement the connections from variables to the clauses *C* of the SAT formula, and are determined, such that seed voltages *ν* or their negations, depending on the specific clause, will attach the output of the seed to a bcTAM OR gate if its potential is greater than or equal to the threshold. These glues are unique to the seed or its negation, and thus, there are 2|*U*| glue pairs. Next, glue rules are determined that connect the outputs of the OR gates to the inputs a single, bcTAM AND gate. There are |*C*| unique glue pairs for input to the AND gate. Thus, the instance of SAT is equivalent to the designed bcTAM, and therefore, their satisfiability is the same. If the SAT formula has a satisfying assignment of variables, then, the bcTAM circuit will have a voltage at the output of the AND gate that is greater than or equal to the threshold, and *vice versa*. This is a valid reduction and thus, bcTAM Satisfiability is NP-Hard.

To clarify the assembly of bcTAM satisfiability, we will demonstrate growth in two example systems, one satisfiable and one not. The first example is a satisfiable formula with variables *u*_*i*_ ∈ *U*:

$$\begin{array}{c} \left(\neg u_{1}\vee u_{2}\right)\wedge u_{3}. \end{array}$$ (15)

**Fig 13:**
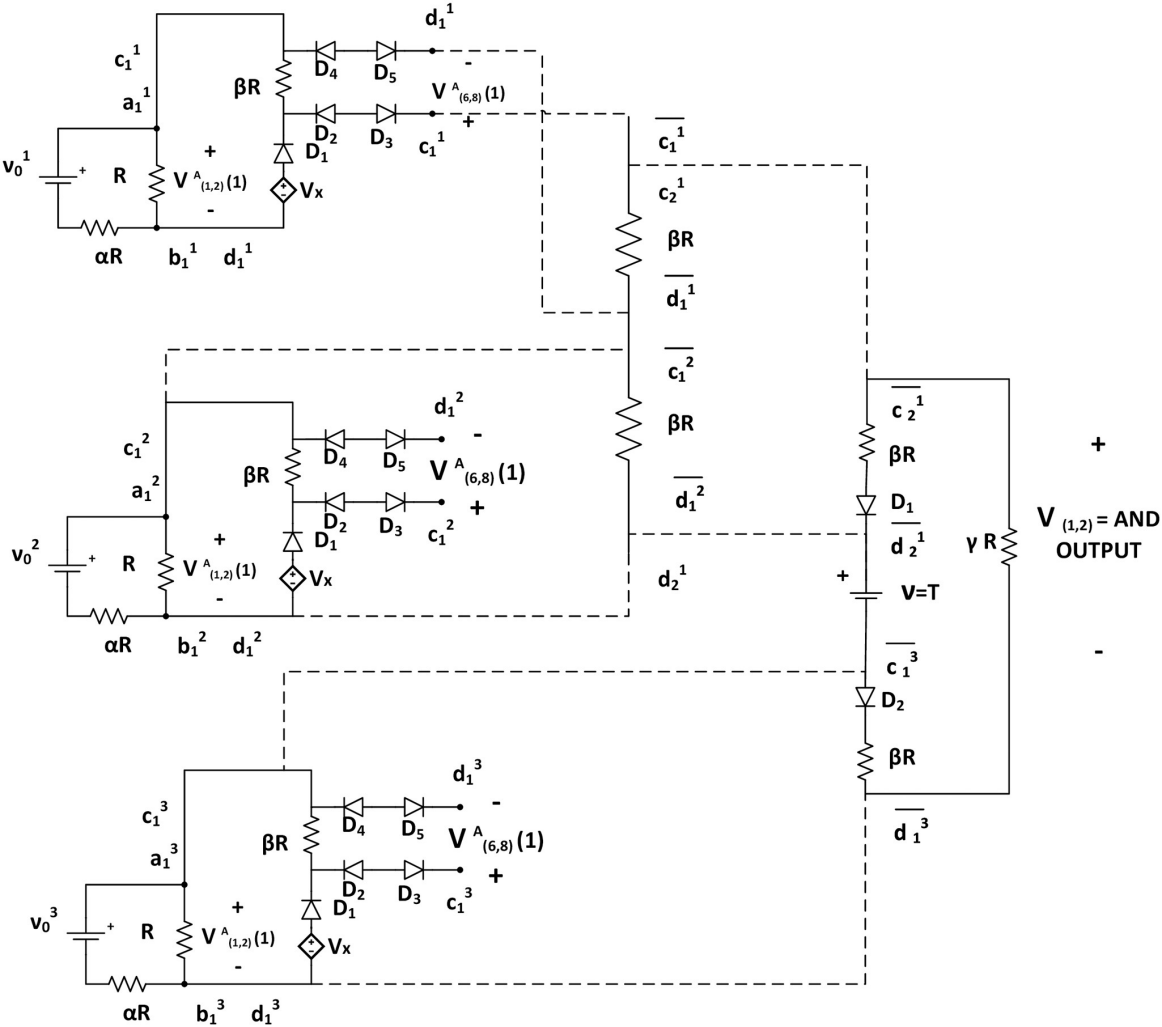
An example of SAT instance mapping to bcTAM SATISFIABILITY problem. Fig. shows the bcTAM implementation of an SAT example represented by Eq 15. Here, the first ladder is terminal, which is analogous to the negation of literal (¬*u*_1_). All other assemblies are still growing. The output of AND tile is >*τ*, and hence, it is a satisfiable circuit. Here, the dotted lines represent the attachment between two tiles using the glues.

This example is analogous to a bcTAM system that has three seed tiles {*s*_1_, *s*_2_, *s*_3_} with input voltages $\left\{\nu_{0}^{1},\nu_{0}^{2},\nu_{0}^{3}\right\}$ respectively that resulted in three ladders. In Fig 13, the NOT output of *s*_1_ and the output of *s*_2_ have glues to bind to the OR gate, representing ¬*u*_1_∨*u*_2_. Then, the output of the OR gate and the output of *s*_3_ have glues to bind to the inputs of the AND gate. The satisfying assignment is *u*_1_ = *FALSE*, *u*_2_ = *TRUE*, and *u*_3_ = *TRUE*. This corresponds to the output of *s*_1_ < *τ*, and the outputs of *s*_2_ and *s*_3_ ≥ *τ*. The output for *s*_1_ is connected from the NOT, which will be ≥ *τ* for $v_{0}^{1}<\tau$. Thus, for the satisfying assignment, both inputs to the OR gate ≥ *τ*, and thus, its output is ≥ *τ*. This with the output of *s*_3_ ≥ *τ* makes the output of the AND gate ≥ *τ*, or TRUE (logic 1). Thus, this bcTAM assembles a satisfiable circuit (Definition 4). The equivalent bcTAM instance is shown in Fig 13.

An example of a non-satisfiable formula is

$$\begin{array}{c} \phi =\neg u_{1}\wedge u_{1}. \end{array}$$ (16)

The analogous bcTAM system of Eq 16 has a single seed tile with source voltage $\nu_{0}^{1}$. From the description of the seed tile (Fig 2), if one output > *τ*, the other output is < *τ* and hence only one output glue pair is activated for attachment. Thus, one input of the AND tile can be connected to one output terminal of the seed, but the other AND input can not be connected to the ladder. Hence, the AND output is < *τ*; and it generates a non-satisfiable circuit.

## Discussion and conclusions

Biological systems have long inspired models of computation, from genetic algorithms to artificial neural networks. Logic gates are a widely accepted model of computation and decision-making [24]. In addition, self-assembly is a core mechanism for biological development and structure formation. In this work, by implementing a computationally complete set of Boolean gates through voltage-controlled self-assembled growth, the bcTAM connects these important ideas. The bcTAM explores how an organism responds in a dynamic environment, *i.e*. variable inputs and threshold. Being able to sense the environment, respond to it, and make a decision, whether conscious or not, is one characteristic of living systems. Moreover, the complexity of this capability is demonstrated by the NP-Completeness of a version of bcTAM assembly.

Variable input voltages (*ν*_0_’s) could have biological relevance as well. They could represent variable sources of energy that produce growth. They could arise as output voltages from sensors, which is common whether the sensor is a neuron or some nonbiological sensor. As the input voltages vary, bcTAM produces different Boolean circuits and, thus, different electric potential distributions at the terminus, as well as throughout the circuit itself. This represents an abstraction of endogenous electric potential distributions, which are produced by membrane potentials. There is increasing evidence that these bioelectric networks influence gene expression and thus, have an important role in embryonic development, including morphogenesis, tissue regeneration, and general biological pattern formation [1–7]. Boolean networks have long been models for genetic regulatory networks [25], and the bcTAM provides an electric analog. In addition, the bcTAM is a system that can decide for itself when the target potential distribution have been achieved through growth by sensing the outputs of logic gates. Thus, with sensory inputs from seed voltages and the randomizing environment represented by the threshold, the bcTAM represents a system that through growth, can sense its environment and make logical inferences about it. This feature is similar to primitive biological mechanisms, such as conformation changes, or organisms, such as physarum. For example, the physarum can explore the paths in a maze, and find the shortest path to the nutrient supplies [26].

Moreover, the bcTAM is implemented with relatively simple circuit components that approximate the DC electric functionality of axons, showing the power inherent in axonal growth. Finally, the relationship between the length of the ladders and the electric potential is known [11, 12], and thus, the input voltages can be determined to a given range based on the length of the ladder. Therefore, the bcTAM provides a new model for biological growth with powerful computational capabilities that might produce further understanding of the role of electric phenomena in biological form and function.

The bcTAM is a resistive network model inspired by biological growth mechanisms, *i.e*. self-assembly, and with circuit components that approximate electrical conduction in axons. For a given set of input parameters, such as a finite number of seed tiles and gate tiles, the bcTAM is a directed system that produces one terminal assembly due to the matching rules criteria. However, if input parameters are variable or multiple gate tiles have the same input glues, it can show non-determinism for growth and result in more than one terminal configuration.

The model performs logical computation with a tile assembly system that is driven by an electric potential. Thus, it provides a new perspective for computation in growing networks of axons, and by extension, the influence of distributions of electric potentials on the development of biological form and function. In the bcTAM, because of its abstraction of electric potential effects on biological growth mechanisms, the resulting networks are amenable to detailed analysis. For example, the range of input potentials to produce given lengths and how the potential changes for each step can be calculated. Thus, from a theoretical perspective, it might produce a better understanding of how Boolean decision-making arises in bioelectric phenomena.

## Supporting information

S1 Appendix Simulation example of OR tile operation. This simulation shows the operation of an OR tile. Here, two ladders grow simultaneously from two seed tiles where *V*_1_ < *V*_2_. Assume the first assembly has a length of two, and the second assembly has a length of three. As long as both of the assemblies or any one of them is growing, the output potential of OR tile (middle tier) is HIGH (> *τ*). When both assemblies are terminals, OR output is LOW. This simulation is conducted on Matlab-Simulink.
(PDF)

S2 Appendix Simulation example of NOT tile operation. This simulation shows the operation of a NOT tile. Here, a terminal ladder has size two. While the ladder is growing, the output potential of NOT tile is LOW (< *τ*). When the ladder is terminal, NOT output is HIGH (> *τ*). Ths simulation is conducted on Matlab-Simulink.
(PDF)

S3 Appendix Simulation example of AND tile operation. This simulation using Matlab-Simulink shows the operation of an AND tile. Here, two ladders are growing simultaneously, where the first assembly has a length of two and the second assembly has a length of three. When both of the assemblies are growing, the output potential of AND tile (middle tier) is HIGH (> *τ*). Otherwise, the AND output is LOW (< *τ*).
(PDF)

S4 Appendix Simulation example of NOR tile operation. This simulation using Matlab-Simulink shows the operation of a NOR tile. Here, two ladders are growing simultaneously from two seed tiles where *V*_1_ < *V*_2_. As long as both of the ladders or any one of them are growing, the output potential of OR tile (middle tier) is LOW (< *τ*). When both assemblies are terminals, NOR output is HIGH (> *τ*).
(PDF)

S5 Appendix Simulation example of NAND tile operation. This simulation using Matlab-Simulink shows the operation of a NAND tile. Here, two ladders are growing simultaneously, where the first assembly has a length of two and the second assembly has a length of three. When both of the assemblies are growing, the output potential of AND tile (middle tier) is LOW (< *τ*). Otherwise, the NAND output is HIGH (> *τ*).
(PDF)
