# Supplementary figures and images for: Logical computation with self-assembling electric circuits
Source: PLoS One. 2022 Dec 7;17(12):e0278033. doi: 10.1371/journal.pone.0278033 (PMC9728908; doi:10.1371/journal.pone.0278033)

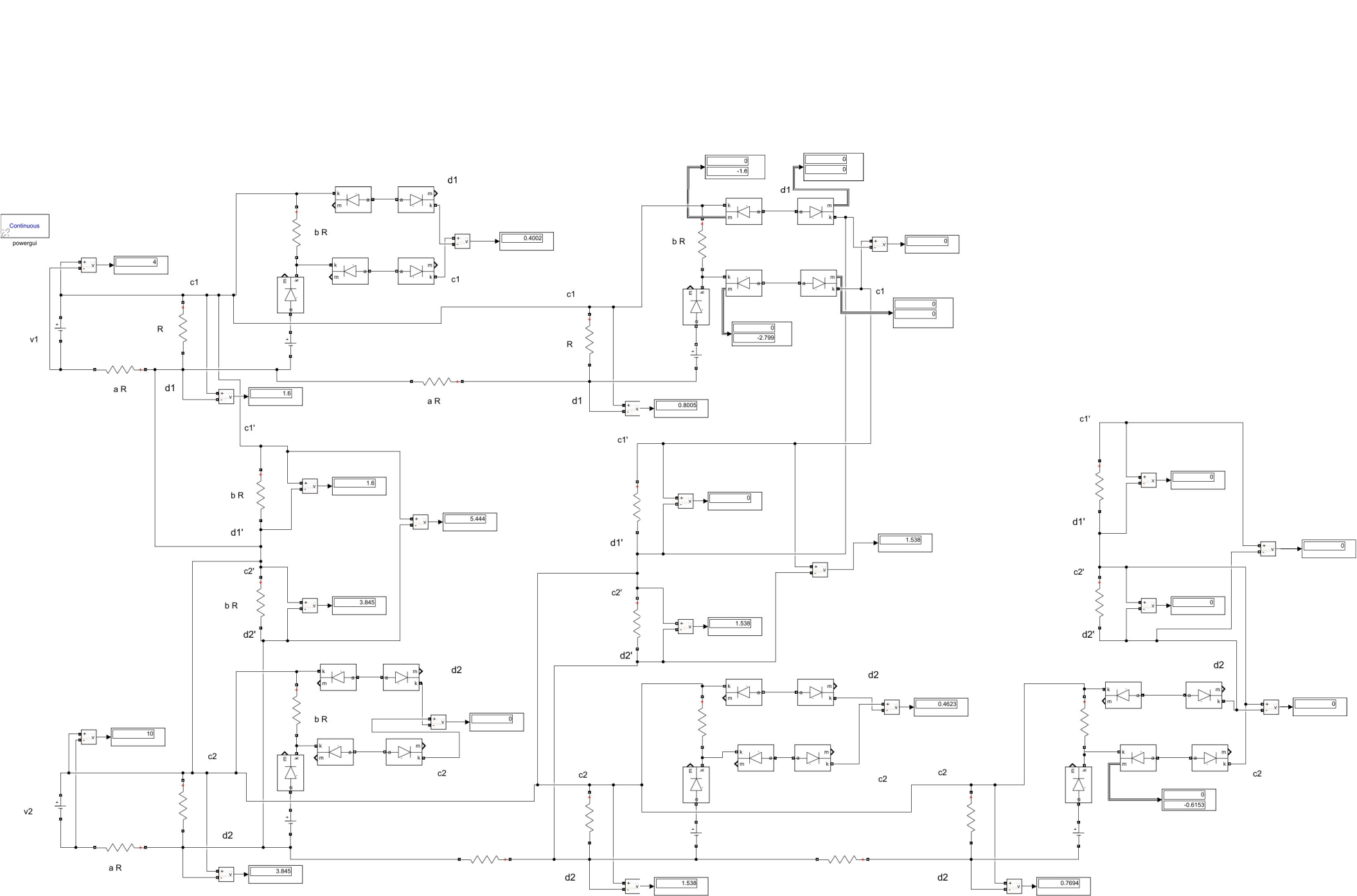

Supplement: S1 Appendix — This simulation shows the operation of an OR tile. Here, two ladders grow simultaneously from two seed tiles where V1 < V2. Assume the first assembly has a length of two, and the second assembly has a length of three. As long as both of the assemblies or any one of them is growing, the output potential of OR tile (middle tier) is HIGH (> τ). When both assemblies are terminals, OR output is LOW. This simulation is conducted on Matlab-Simulink. (PDF) [file pone.0278033.s001.pdf]

Continuous  
powergui

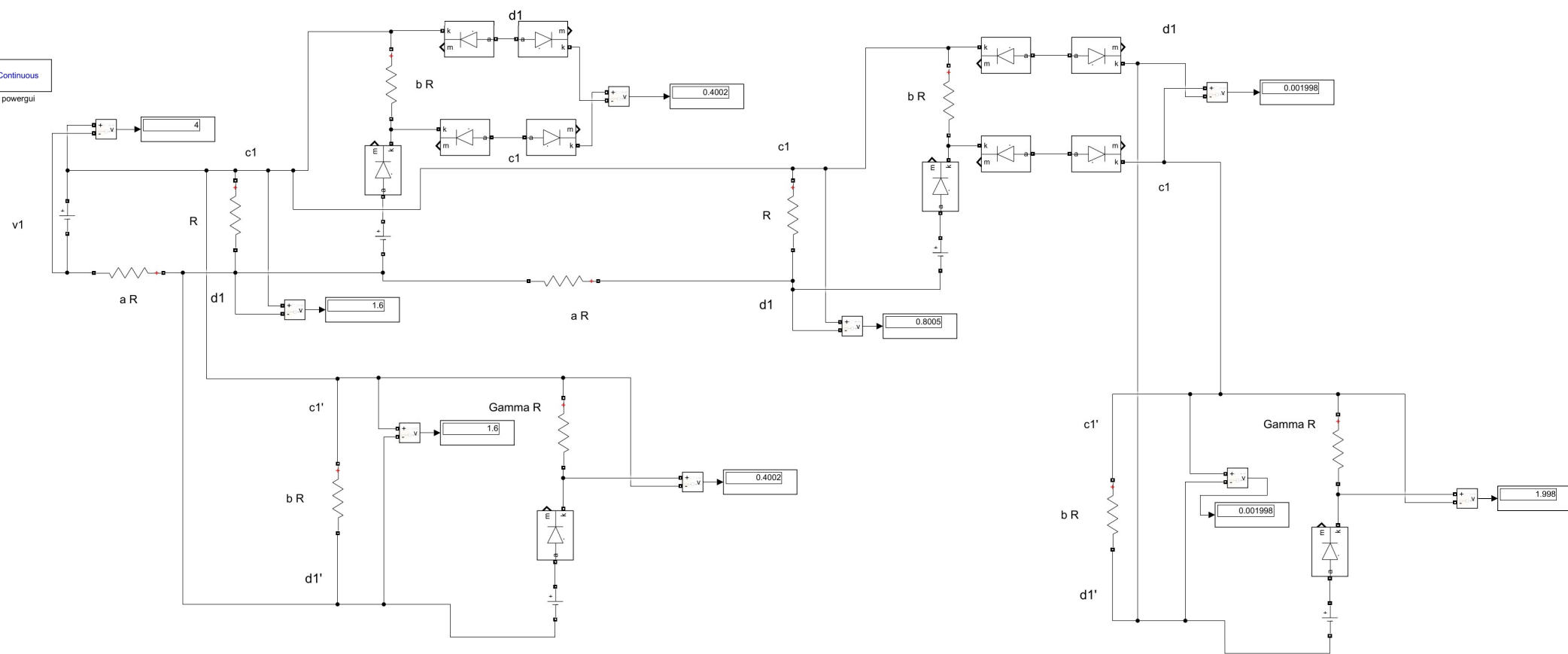

Supplement: S2 Appendix — This simulation shows the operation of a NOT tile. Here, a terminal ladder has size two. While the ladder is growing, the output potential of NOT tile is LOW (< τ). When the ladder is terminal, NOT output is HIGH (> τ). Ths simulation is conducted on Matlab-Simulink. (PDF) [file pone.0278033.s002.pdf]

Continuous  
powergui

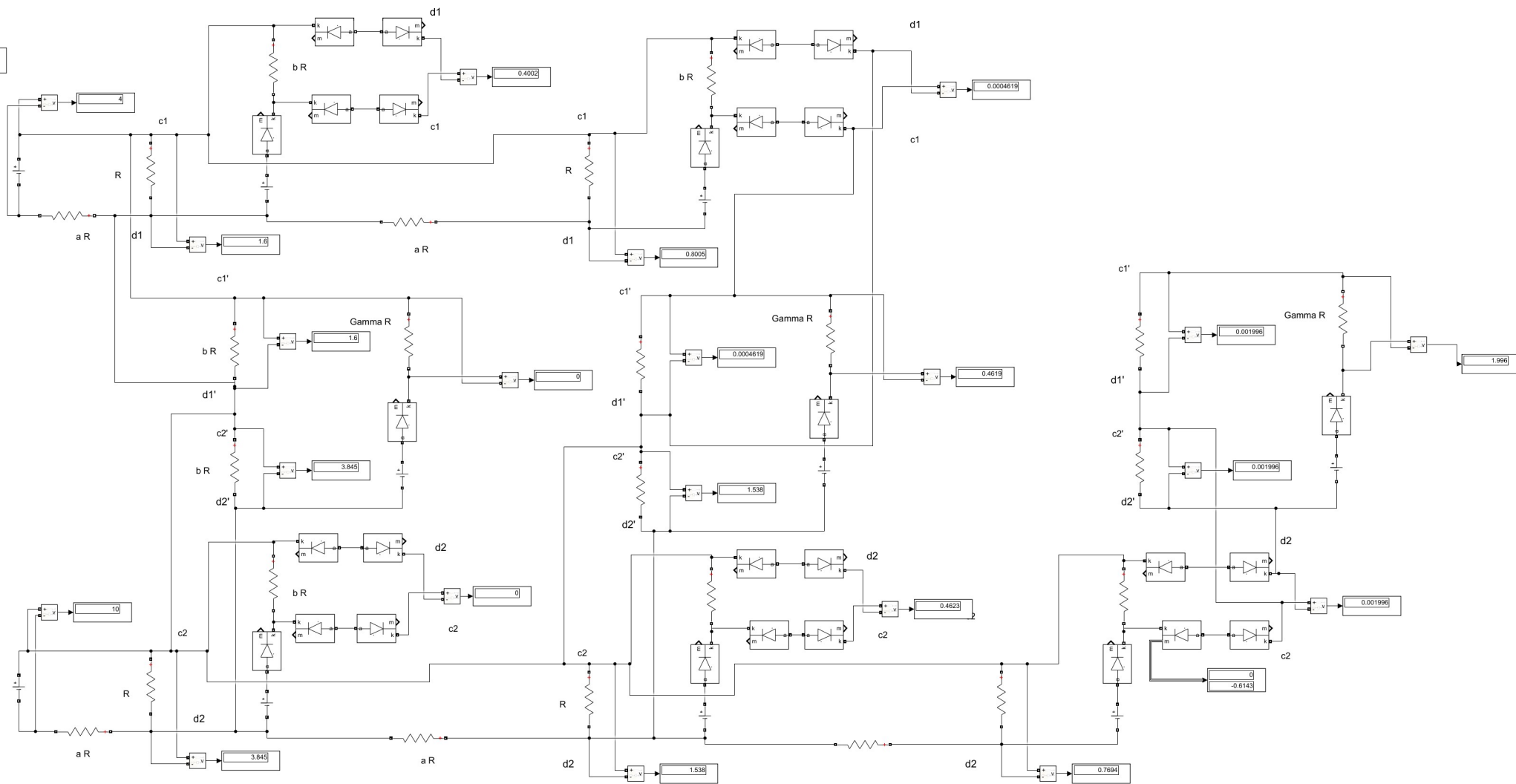

Supplement: S4 Appendix — This simulation using Matlab-Simulink shows the operation of a NOR tile. Here, two ladders are growing simultaneously from two seed tiles where V1 < V2. As long as both of the ladders or any one of them are growing, the output potential of OR tile (middle tier) is LOW (< τ). When both assemblies are terminals, NOR output is HIGH (> τ). (PDF) [file pone.0278033.s004.pdf]

Continuous  
powergui

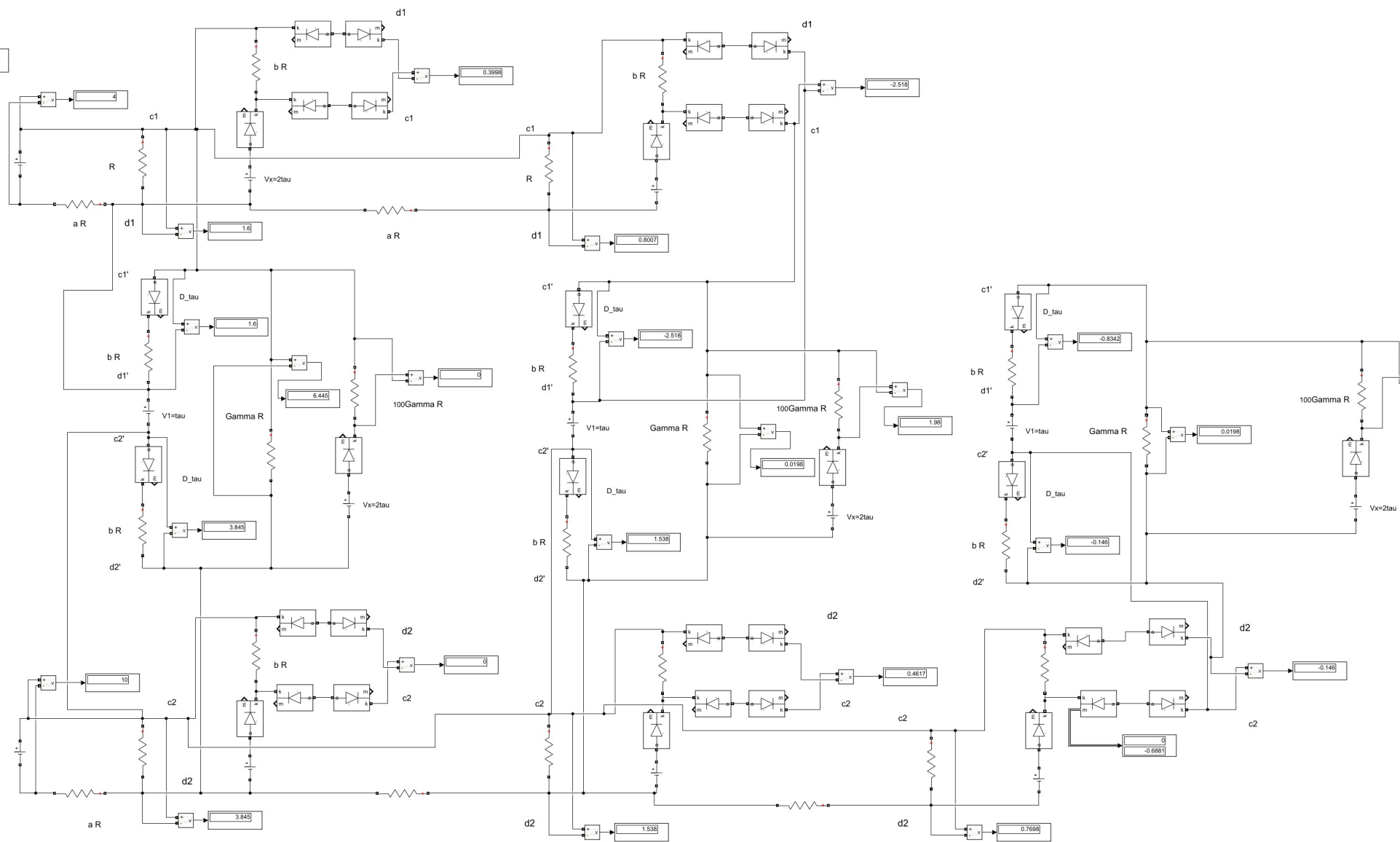

Supplement: S5 Appendix — This simulation using Matlab-Simulink shows the operation of a NAND tile. Here, two ladders are growing simultaneously, where the first assembly has a length of two and the second assembly has a length of three. When both of the assemblies are growing, the output potential of AND tile (middle tier) is LOW (< τ). Otherwise, the NAND output is HIGH (> τ). (PDF) [file pone.0278033.s005.pdf]
