# Supplementary material for: Logical computation with self-assembling electric circuits
Source: PLoS One. 2022 Dec 7;17(12):e0278033. doi: 10.1371/journal.pone.0278033 (PMC9728908; doi:10.1371/journal.pone.0278033)
Supplement: S3 Appendix — This simulation using Matlab-Simulink shows the operation of an AND tile. Here, two ladders are growing simultaneously, where the first assembly has a length of two and the second assembly has a length of three. When both of the assemblies are growing, the output potential of AND tile (middle tier) is HIGH (> τ). Otherwise, the AND output is LOW (< τ). (PDF) [file pone.0278033.s003.pdf]

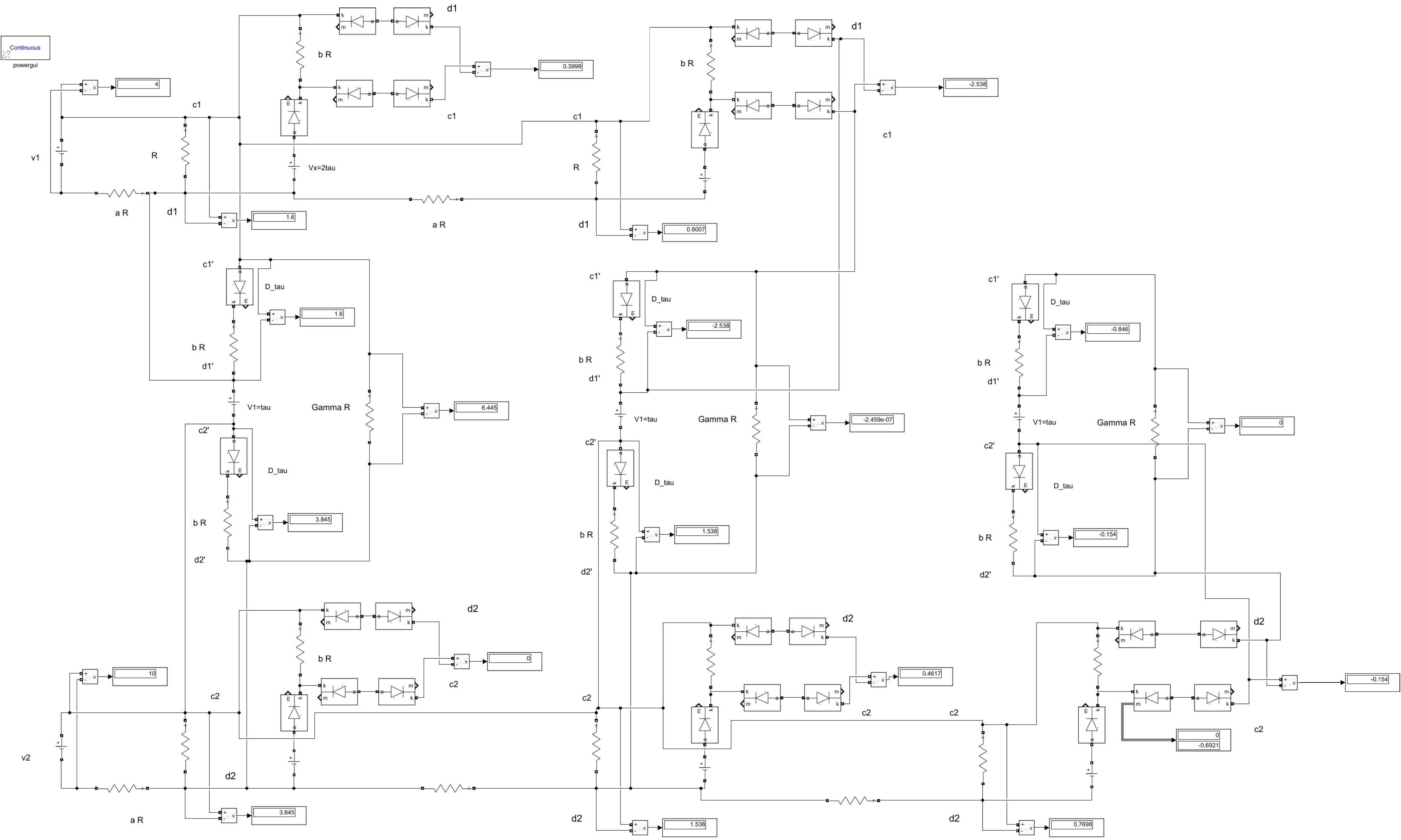

Continuous
powergui
d1
b R
0.3998
4
c1
v1
R
Vx=2tau
a R
d1
1.6
c1'
D_tau
1.6
b R
d1'
V1=tau
Gamma R
6.445
c2'
D_tau
b R
3.845
d2'
d2
b R
0
10
c2
c2
v2
d2
a R
3.845
d1
b R
-2.538
c1
c1
R
a R
d1
0.8007
c1'
D_tau
-2.538
b R
d1'
V1=tau
Gamma R
-2.459e-07
c2'
D_tau
b R
1.538
d2'
d2
0.4617
c2
c2
c2
d2
1.538
c1'
D_tau
-0.846
b R
d1'
V1=tau
Gamma R
0
c2'
D_tau
b R
-0.154
d2'
d2
-0.154
0
-0.6921
c2
d2
0.7698
